# Parallel engineering of environmental bacteria and performance over years under jungle-simulated conditions

**DOI:** 10.1371/journal.pone.0278471

**Published:** 2022-12-14

**Authors:** Yonatan Chemla, Yuval Dorfan, Adi Yannai, Dechuan Meng, Paul Cao, Sarah Glaven, D. Benjamin Gordon, Johann Elbaz, Christopher A. Voigt

**Affiliations:** 1 Synthetic Biology Center, Department of Biological Engineering, Massachusetts Institute of Technology, Cambridge, Massachusetts, United States of America; 2 School of Molecular Cell Biology & Biotechnology, Faculty of Life Science, Tel Aviv University, Tel Aviv, Israel; 3 The Broad Institute of MIT and Harvard, Cambridge, Massachusetts, United States of America; 4 Center for Bio/Molecular Science and Engineering, Naval Research Laboratory, Washington, DC, United States of America; Universidad Nacional Autonoma de Mexico, MEXICO

## Abstract

Engineered bacteria could perform many functions in the environment, for example, to remediate pollutants, deliver nutrients to crops or act as in-field biosensors. Model organisms can be unreliable in the field, but selecting an isolate from the thousands that naturally live there and genetically manipulating them to carry the desired function is a slow and uninformed process. Here, we demonstrate the parallel engineering of isolates from environmental samples by using the broad-host-range XPORT conjugation system (*Bacillus subtilis* mini-ICE*Bs*1) to transfer a genetic payload to many isolates in parallel. *Bacillus* and *Lysinibacillus* species were obtained from seven soil and water samples from different locations in Israel. XPORT successfully transferred a genetic function (reporter expression) into 25 of these isolates. They were then screened to identify the best-performing chassis based on the expression level, doubling time, functional stability in soil, and environmentally-relevant traits of its closest annotated reference species, such as the ability to sporulate and temperature tolerance. From this library, we selected *Bacillus frigoritolerans* A3E1, re-introduced it to soil, and measured function and genetic stability in a contained environment that replicates jungle conditions. After 21 months of storage, the engineered bacteria were viable, could perform their function, and did not accumulate disruptive mutations.

## Introduction

Complex and unique communities of bacteria live in soil, on building materials, on equipment and vehicles and are associated with humans, plants, and animals [1]. Functions could be added to these environments by introducing living bacteria engineered to carry the associated genetics [2–4]. Model strains, such as *Escherichia coli* are easy to engineer, but are rapidly outcompeted by native species within a few weeks [5]. Evolving *E*. *coli* for a particular environment or using alternative model chasses (*e*.*g*., *Pseudomonas putida* or *Bacillus subtilis*) can improve persistence, but it can also introduce a foreign species into the ecosystem, which can create a regulatory hurdle. Moreover, successful colonization is highly variable and unpredictable across field conditions and the composition of the microbiota [4, 6–10]. Alternatively, the function could be introduced into resident bacteria using either *in situ* techniques [11, 12] or by isolating, engineering and re-introducing the bacteria [2, 3, 13, 14]. The challenge is identifying which of the sometimes thousands of native bacteria is the most suitable host to carry this function.

There are many examples of functions carried by living organisms that have been or can be deployed to the field. Living biosensors have been used for environmental monitoring of many compounds. Examples include engineered *E*. *coli* TNT sensors that have been deployed encapsulated in alginate beads to tackle the grand challenge of mine detection and clearance [4, 15], and plants have been engineered to monitor levels of the opioid fentanyl [16]. Field bioremediation of pollutants has been conducted using engineered *P*. *putida* cloned with phenol degrading operons and released to degrade pollution caused by a major shale mine fire in Estonia [3]. Microbes can also be engineered to remediate and recycle mine-seepage pollutants like selenate [17], oil spills, or plastic waste. In agriculture–chassis are used for augmented nitrogen-fixing biofertilizers [18] and crop protection and growth [19], and in medicine–commensal microbes can be engineered for skin [13] or gut probiotics [20] to sense or treat diseases. Furthermore, fieldable functions include self-assembling [21] and self-healing [22, 23] materials, rare earth biomineralization, and biomining [24, 25].

Different species vary in their ability to perform an engineered function, deal with the additional burden of carrying it, and re-colonize, grow and persist in the environment. For example, if the new function is the production of a chemical or material, some species may harbor higher concentrations of precursor metabolites [26, 27], and simply expressing heterologous proteins imparts a growth burden that may differ between species [28–30]. In the target environment, species will differ in their growth rates, synergistic and antagonistic interactions with the native microbiota, and react differently to fluctuations in conditions and stresses [31, 32]. The ability and propensity to form spores and rates of re-germination also impact long-term persistence [22, 33, 34]. Finally, genetic stability differs across species; some may more rapidly evolve than others to mutate or excise the payload to reduce its burden on the cell [35–37].

Previously, it has been challenging to introduce the same function across a large panel of species newly isolated from the environment and perform a side-by-side comparison of performance. To facilitate cross-species screening, we previously developed a conjugation strain (XPORT) that can transfer a genetic “payload” to many species in parallel, including undomesticated strains [38]. XPORT uses machinery from a *Bacillus subtilis* integrative and conjugative element (ICE) that we engineered to make transfer inducible, prevent future propagation after the initial event, minimize the amount of conjugation DNA that transfers with the payload, and enable recipient isolation using D-alanine auxotrophy [39, 40]. This method was previously used to transfer an IPTG sensor to 35 known species isolated from soil and the human microbiome and to compare the response functions across species [40]. It also was previously used to measure the production of the black pigment melanin across species, including contaminants of an industrial process, to identify production strains to be grown within living building materials [38, 41]. This involved balancing the titer achieved under laboratory conditions with the growth rate in the material during the production process and interference with the fungus used for production in order to find a balanced strain.

In this manuscript, we report the use of XPORT to transfer a function to bacteria isolated from seven soil and water samples from the surface and underground of two sites in Israel. We designed a construct to overexpress green fluorescent protein (GFP) and used XPORT to transfer it into 25 previously unknown isolates of *Bacillus* and *Lysinibacillus*. Of these, *Bacillus frigoritolerans* A3E1 was selected for further study because of its high expression level and rapid doubling time. To demonstrate the use of the engineered strain (GFP+) for tagging, we mixed it with soil and showed that it could be imaged on textiles using a low-cost blue flashlight. The GFP+ strain was evaluated for performance and persistence under realistic and stressful conditions in soil in a contained “jungle room” maintained by Naval Research Laboratories that included plants, rain, and UV exposure and in a storage room for nearly two years. The strain was shown to persist and maintain function for over two weeks in the jungle room while it retained its function throughout the experiment under storage conditions. After 21 months, the strain was re-sequenced and was found to contain only 6 mutations in genes, none of which were within the genetic payload. This work demonstrates the process of isolation, transfer of DNA, and screening as a means to rapidly and reliably introduce a new function into the field.

## Results

### Isolation of bacteria from soil and water samples

**Fig 1:**
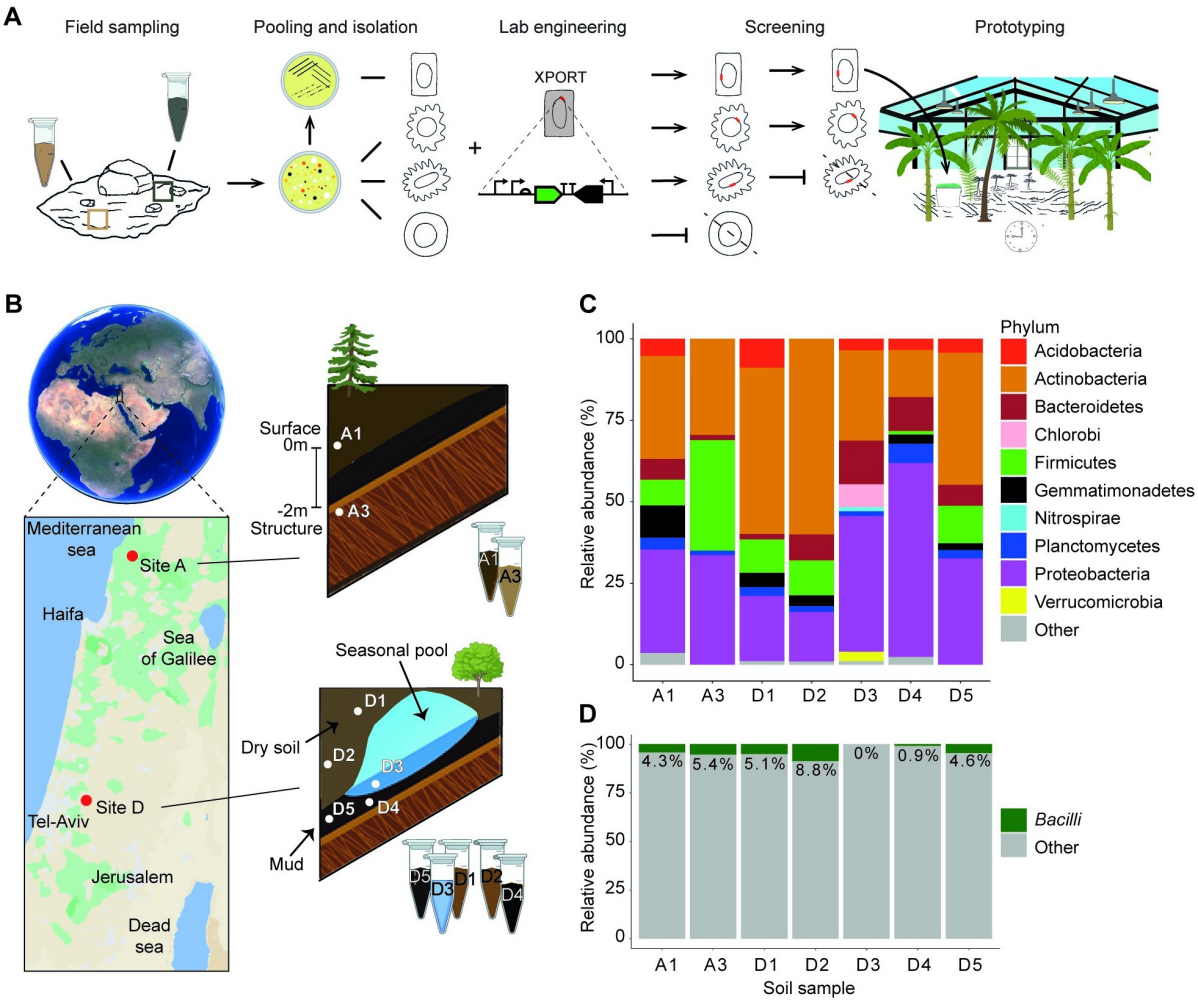
Overview of prototyping strategy and soil samples used. **(a)** A stepwise schematic of the pipeline developed in this study. **(b)** Soil sampling locations and types. Approximate locations and the names of the sampling sites are marked in red over a map of the southeastern Mediterranean shore. White dots represent sample types and names. **(c)** 16S rDNA gene sequencing analysis data of soil samples showing the Phyla-level relative abundance. The ‘Other’ category includes all phyla with relative abundance <1%. **(d)** Metagenomic data showing the relative abundance of the Bacilli class in soil samples.

We collected seven soil and surface water samples from two sites in Israel (Fig 1B). Site A, located in Upper Galilee, in the vicinity of Zar’it, was sampled on a rainy day on December 27, 2018. In these typical Mediterranean hills (elevation of 500m), the climate is seasonal, average summer high of 33°C and a winter low of 4°C, with 64% humidity. Almost all the 653 mm of yearly precipitations occur in winter. The silt loam soil types in this location are Cenomanian and Turonian limestone, with typical Mediterranean flora and fauna with little human activity. From site A, two samples were taken, one from the surface soil (A1) and the other from soil excavated close to an approximately 2-meter-deep human-built structure (A3). Site D, located in the Sharon plain of central Israel, on a training ground of the Sirkin Base, was sampled on March 13, 2019. The Mediterranean climate at this site is warm, with an average summer high of 32°C and winter low of 9°C, and 67% humidity. During the year, there is low average precipitation of 314 mm, most of it during the winter. The silt loam soil in this site was alluvial, and the area is urban with many small buildings, some vegetation, and significant human presence and activity. From this site, five samples were taken from different local environments: two surface samples dug from approximately 50 cm depth (D1, D2), and three samples from inside a human build underground shaft: one wet (D4) and one mud (D5) soil samples, and one water sample (D3) from a small seasonal pool.

16S rRNA gene sequencing analyses were performed to assess the bacterial species diversity in each sample (Fig 1C). The results show distinct bacterial phyla composition, similar to previously reported soil microbiomes [42], and in samples A3 and D3-D5, which were taken from in or near human-built structures, the compositions were similar to urban structures [1], where Firmicutes and Proteobacteria are enriched. In the soil samples, Bacilli-class species ranged between 0.9–8.8% of total bacterial abundance (Fig 1D). In the water sample (D3), no Bacilli were detected. However, we could still mate XPORT with *Lysinibacillus* recipients from this sample. This is likely due to a lack of sufficient metagenomic sequencing depth to detect these low abundance *Lysinibacillus* species.

Extraction of bacterial isolates from soil samples was performed and the isolates were prepared for conjugation using three methods (S1 Fig in S1 File). The first method, “in culture,” allowed potential mating with all the bacteria present in the soil sample. This was done by incubating the soil in water at 37°C, followed by incubation with the donor strain and mating (Methods). The second method, “pooled colonies,” allowed mating with a subset of bacteria extracted from samples. This was done by incubation of sample extracts in LB media, followed by plating the extracted bacteria on LB agar plates. Next, we pooled the hundreds of colonies from the plates and performed mating on the mixed culture. The same protocol was followed for the third method, “isolated colony,” except, instead of pooling, two isolated colonies were picked from samples A1 and A3, and individually mated.

Each method has advantages and limitations. Method 1 has the largest potential recipient diversity but lacks sensitivity when the abundance of recipients is too low in the original sample. Method 2 loses diversity but allows for enrichment of isolated strains’ abundance, which increases conjugation frequency. Method 3 is limited to a low number of isolates, but unlike methods 1 and 2, which use bacterial mixtures for mating, it allows distinction and preserves the wildtype isolates used for mating.

### Payload design and genomic conjugation into isolates and chassis screening

**Fig 2:**
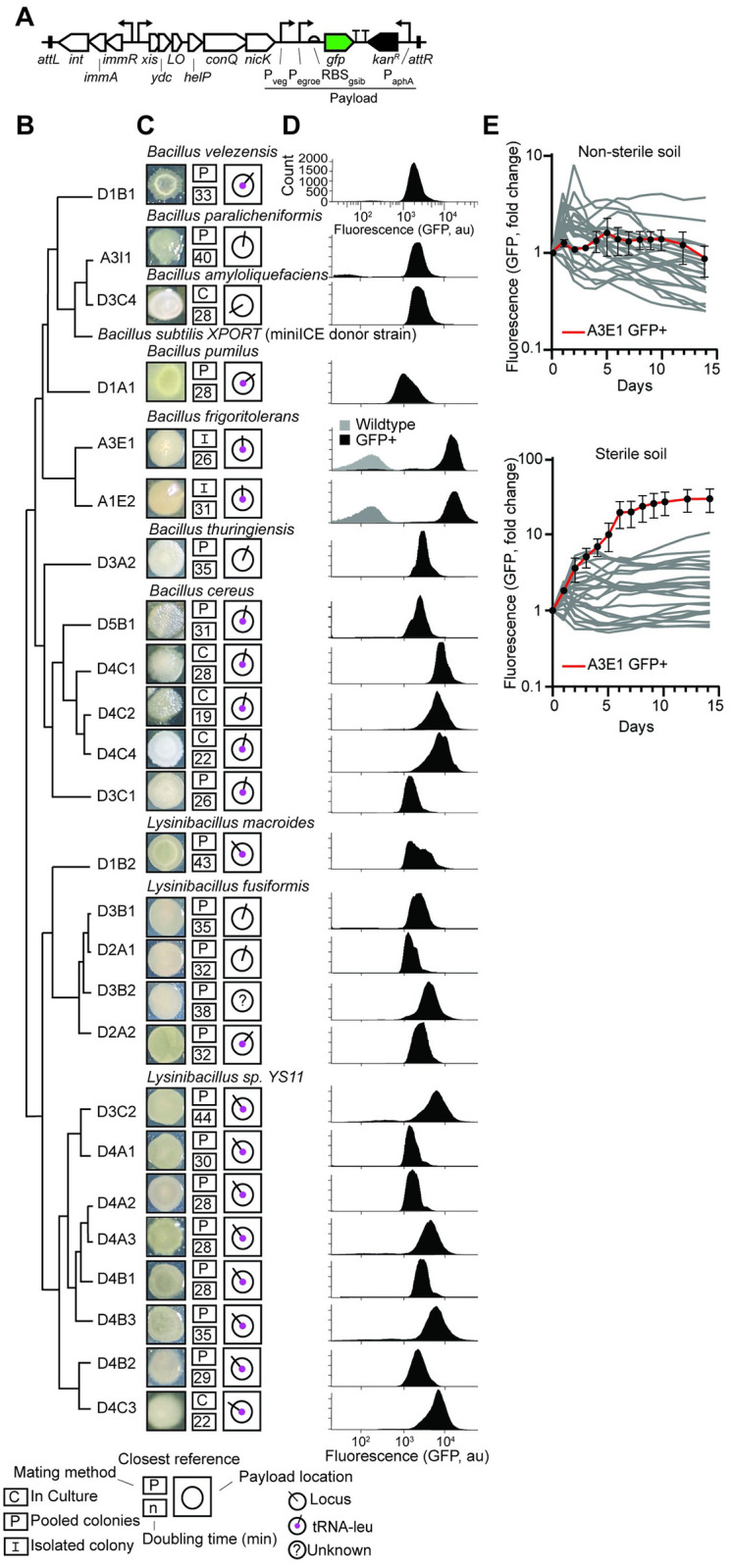
Rapid and parallel screening of engineered undomesticated bacteria from soil samples. **(a)** Genetic map of the mini-ICE*Bs*1 cassette conjugated from the donor *B*. *subtilis* XPORT strain to recipient strains. **(b)** Phylogenetic tree based on 16S sequences (methods) describing the isolated and engineered undomesticated soil strains. Names of undomesticated isolates reflect the site and soil sample they were isolated from (e.g., D1B1 was isolated from site D1 in sample B1). **(c)** Characterization of colony morphology on agar (colony diameter in images ranges between 0.2–0.5 cm), the methods used to prepare recipient cells for mating (P–“Pooling,” C–“Culture,” I–“Isolated”; methods), doubling time of isolate (in minutes), and genomic conjugation loci of the mini-ICE*Bs*1 cassette identified through whole-genome sequencing of each isolate. **(d)** Representative flow cytometry measurements of the fluorescence intensity of isolated and engineered bacteria populations. The results were replicated independently three times **(e)** Fluorescence stability over time of engineered strains in non-sterile and sterile soils incubated at 24°C.

A genetic payload that contains a gene encoding green fluorescent protein (*gfp*) as a reporter and the kanamycin resistance gene (*kan*) as a selectable marker was inserted into the mini-ICEI*Bs*1 cassette in XPORT (Fig 2A) [38]. To express GFP, two strong *B*. *subtilis* tandem constitutive promoters (*P*_*veg*_ and *P*_*egroe*_) were selected that were known to have broad host specificity [43, 44]. The *kan* resistance gene was constitutively expressed in the antisense strand using its native *aph(3’)* promoter and RBS. A double terminator was placed between the *gfp* and *kan* genes to avoid transcriptional interference.

XPORT was used to conjugate the payload into strains isolated from soil. Mating experiments between the donor and recipient cultures (derived through the three methods, above) were performed by mixing and spotting each donor-recipient culture pair separately (Methods). After incubation and donor-recipient conjugation of the payload, each resulting sample was plated on kanamycin-selective LB agar plates. The plates lacked D-alanine, providing a negative selection against the XPORT donor. The Site D soil samples were subjected to either Method 1 or 2, and this process resulted in hundreds of colonies that grew under kanamycin selection. The Site A samples, subjected to methods 2 and 3, exhibited smaller colony diversity observed by eye, with only several colony types that could grow under kanamycin selection.

We picked 25 colonies from all sampling locations and conjugation methods that were fluorescent due to GFP expression. The phylogenies of the 25 colonies were determined by 16S rDNA sequencing and phylogenetic analysis (Fig 2B) (Methods). All were part of the *Bacillaceae* family and belonged to either the *Bacillus* or *Lysinibacillus* genera. This is likely due to the higher *B*. *subtilis* XPORT conjugation efficiency for its family [40] and the use of promoters derived from *B*. *subtilis* to express GFP.

Whole-genome sequencing (WGS) was performed on all 25 isolates and was used to determine the genomic locus in each isolate where the mini-ICE*Bs*1 payload was inserted (Fig 2C and S1 Table in S1 File). In 19/25 isolates, the conjugation locus was adjacent to tRNA-leu, as expected when using the original mini-ICE*Bs*1 cassette [40]. However, in five cases, conjugation occurred at other genomic loci, and in one case, the genomic locus could not be detected, indicating that it may be carried as a plasmid [40]. The pairwise genetic distance between the isolates isolated in this study and their closest annotated references varied between 1% to 11% of the genomes (S2 Fig in S1 File).

The doubling time of each isolate in LB media at 37°C ranged between 19–44 minutes, which is typical of Bacilli (Fig 2C) [45]. GFP expression was measured for each strain using flow cytometry, with a 40-fold difference in the median fluorescence across isolates (Fig 2D) (Methods). The highest expression levels were obtained by *B*. *frigoritolerans* A1E2 and A3E1, isolated from Site A.

We then assessed the capability of each isolate to maintain a constant cell concentration and GFP expression in soil over time. To do this, a fixed number of cells were mixed in sterile and non-sterile soil from site D. Fluorescence measurements were conducted daily for 14 days while incubating soils at 24°C and 90% humidity (Fig 2E and S3 Fig in S1 File). Across all strains, the total fluorescence was higher in the sterile soil as compared with the nonsterile soil. In nonsterile soil, the isolates’ total function ranged between a 4-fold increase (*B*. *cereus* D3C1) and a 4-fold decrease (*Lysinibacillus* sp. D4C3), *B*. *frigoritolerans* A3E1 activity remained stable throughout the experiment around the same levels as day 1. In sterile soil, the isolates’ fluorescence at day 14, ranged between a 30-fold increase (*B*. *frigoritolerans* A3E1) and a 2-fold decrease (*Lysinibacillus* sp. D4A3).

*Bacillus frigoritolerans* A3E1 was selected because of the high GFP expression, fast growth rate, and the maintenance of heterologous expression in sterile and non-sterile soil over time. Its closest reference strain (*B*. *frigoritolerans* DSM-8801; 96.5% DNA sequence identity), which was originally isolated in Morocco from warm and arid soil [46], has demonstrated soil persistence of more than 42 days [47], and has been suggested for field applications such as organophosphate bioremediation [47, 48] (S4 Fig in S1 File). This obligate aerobe, spore-forming bacterium shows high tolerance to temperature variation, salinity, osmotic stress, and radioactivity [48, 49]. In addition, *B*. *frigoritolerans* was shown to enhance plant salt and drought tolerance [50] and could kill insect pests [51]. The 16S rDNA analysis was used to quantify the abundance of *B*. *frigoritolerans* in sample A3, the only one in which it was detected, where it accounted for 0.75% of all total bacterial species that were measured (S5 Fig in S1 File). In the genome of the isolated strain (5.855 Mbp), we identified several genes which were missing from its closest reference genome (5.460 Mbp; Table 1). These included potential members of degradation pathways for atrazine—a common herbicide, styrene, and toluene, and siderophore nonribosomal peptide biosynthesis genes.

**Table 1:** Unique biosynthetic genes of *B*. *frigoritolerans* A3E1 compared to its reference DSM (8801).

Pathway^a^	Gene	Gene ID in Novel assembly	KEGG pathway #
Atrazine degradation	Urease subunit beta	FRI_000110T0	00791
	Urease subunit gamma	FRI_000111T0	
	Allophanate hydrolase	FRI_002780T0	
	urea carboxylase	FRI_002781T0	
Toluene degradation	Catechol 1,2-dioxygenase	FRI_003103T0	00364
	Muconate cycloisomeras	FRI_003106T0	
Biosynthesis of siderophore group nonribosomal peptides	2,3-dihydro-2,3-dihydroxybenzoate dehydrogenase	FRI_001107T0	01053
	2,3-dihydroxybenzoate-AMP ligase	FRI_001109T0	
	Amino acid adenylation domain containing protein	FRI_001111T0	
Styrene degradation	Catechol 1,2-dioxygenase	FRI_003465T0	00643

^a^. Based on homology search against the KEGG database (with minimum coverage of 70%).

### Textile provenance using GFP+ B. frigoritolerans A3E1

**Fig 3:**
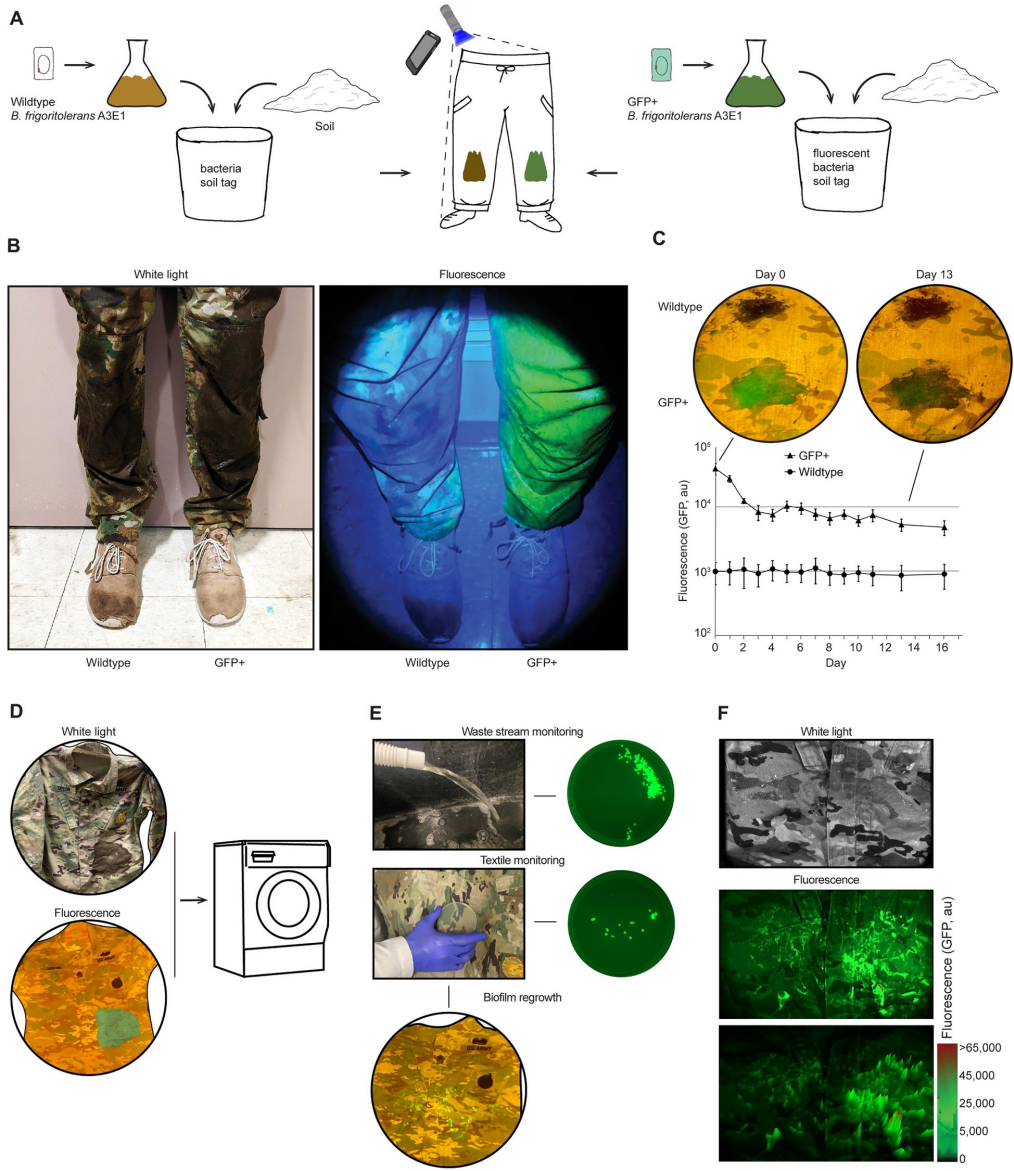
Applications for bacteria-infused soil on textiles. **(a)** Schematic of the application **(b)** Cell-phone pictures of trousers after smearing with WT (right leg) and GFP+ (left leg) *B*. *frigoritolerans* A3E1, under white light (left) and fluorescence imaging using Blue LED flashlight (450 nm) and 505 nm filter taped to the camera (right). **(c)** The stability of GFP+ *B*. *frigoritolerans* A3E1 fluorescence applied over time measured using a fluorescence imager to quantify the mean fluorescence of an area of the textile. Error bars represent the standard deviation of all pixels within the area. Inset represents the fluorescent images of the textile tagging, taken each day using a cell-phone camera. **(d)** Representative white light and fluorescence images, using a cell phone camera, of the GFP+ *B*. *frigoritolerans* A3E1 smeared textile before machine washing **(e)** Monitoring of smeared bacteria after washing (top) in the washing machine’s waste stream. The waste stream was sampled and spread on selective agar plates, which were incubated overnight and imaged in a fluorescence imager. (middle) Monitoring by textile imprinting using selective agar plates pressed upon the textile, incubated overnight, and imaged in a fluorescence imager. (bottom) biofilm regrowth by incubation with selective media on the washed textile fluorescence image using a cell-phone camera **(f)** Imaging of biofilm regrowth on textile in a fluorescent imager. (top) white light, (middle) fluorescence image, (bottom) mountain plot of the fluorescence image.

A potential application for engineering soil bacteria is to track mud smeared on personnel or vehicles that move through a protected area. Such applications can be used, for example, to protect conservation areas against poaching or other damaging activities. As a simple demonstration, we grew cultures of GFP+ and wildtype (WT) *B*. *frigoritolerans A3E1*, and mixed them with autoclaved soil. The resulting bacteria-infused mud was stored at room temperature for a day. Then, the mud was spread on trousers (nylon cotton ripstop fabric), one side with mud infused with wildtype bacteria and the other side with GFP+ bacteria (Fig 3A). The fluorescent imaging was performed in a darkened room using a cell phone camera covered with a 505 nm filter taped over the lens. The light source was a commercially available blue LED flashlight (450nm). The fluorescent bacteria were readily detectable in still images and video (Fig 3B). Next, the detectability of the signal over time was tested over the course of 16 days (Fig 3C). The results show that the signal was visible throughout the experiment.

We also smeared a shirt (nylon cotton ripstop fabric) with 10 g of sterile soil infused with GFP+ *B*. *frigoritolerans* A3E1 to evaluate if the bacteria would still be detectable after washing (Fig 3D). The textile was machine washed for 10 minutes (1 mL detergent / 1 L water) in 60°C hot water (U.K Department of Health guidelines for disinfecting foul and used and infected linen) [52], or 20°C cold water (field conditions). After laundry, the bacteria-infused mud stains were completely unobservable, and no fluorescence was detectable. However, when the wastewater stream was sampled, by directly plating wastewater on selective LB agar plates, GFP+ *B*. *frigoritolerans* A3E1 were readily detected (Fig 3E). This resulted in 4.5 ± 1.3 and 420 ± 127 CFUs/mL for the hot and cold water, respectively. In addition, we were able to detect bacteria from the washed textile directly. One hour after laundry, we pressed selective agar plates against the uniform to imprint residual bacteria from the textile (Fig 3E). This resulted in 3.3 ± 2.5 and 27 ± 3.6 CFUs/imprint of the GFP+ bacteria for the hot and cold water, respectively. The results indicate that the bacteria in the textile can be detected after washing using either the textile itself or a facility waste stream. Lastly, we were able to regrow GFP+ *B*. *frigoritolerans* A3E1 on the uniform after washing by covering areas of the shirt with selective LB agar containing kanamycin and incubating the washed shirt for 48 hours at 37°C (Fig 3E). The biofilm was readily detectable by fluorescence, and the biofilm covered large areas of the fabric beyond its original smear area with variable levels of fluorescence (Fig 3F), and it continued to grow when it was supplemented with additional LB.

### Long-term testing of engineered bacteria under simulated field conditions

**Fig 4:**
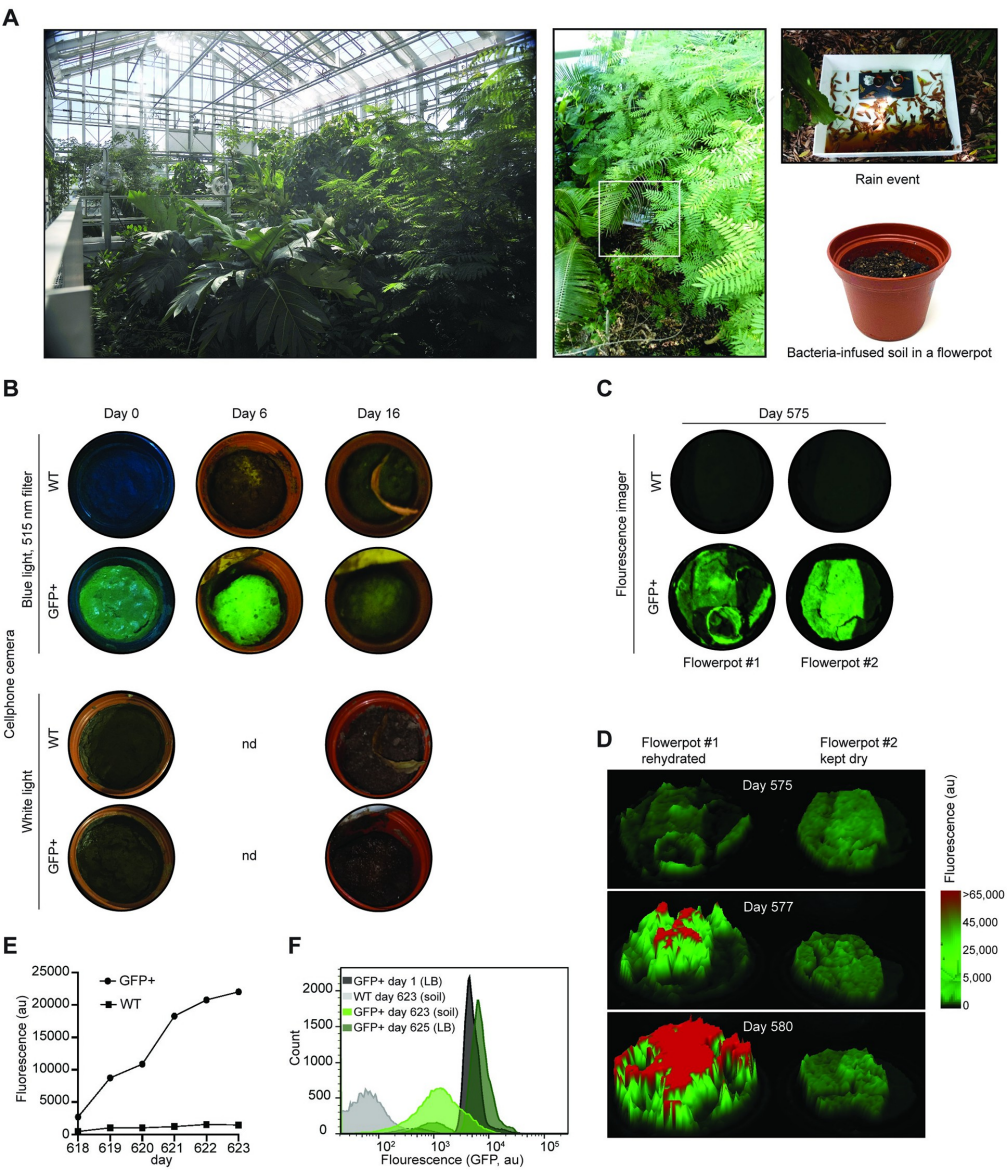
Bacterial prototyping in replicated environments. **(a)** Jungle room photos, showing the location of the *B*. *frigoritolerans* A3E1 infused soil samples in flowerpots on a rain event on day six. **(b)** Simulated environment survival experiment results. Cell-phone pictures of Wildtype and GFP+ *B*. *frigoritolerans* A3E1 containing pots under Blue LED flashlight illumination (450 nm) and 505 nm filter (top) and white light (bottom) at three time-points during the experiment. **(c)** Fluorescent images of GFP+ and WT containing soil flowerpots on day 575 of long-term storage **(d)** Representative comparison between rehydrated (left) and dry (right) GFP+ containing pots and during the rehydration process. Mountain plots describe GFP fluorescence intensity. The red color represents over-exposure. The experiment has been replicated twice. **(e)** Quantification of the soil-surface mean fluorescent levels during the rehydration experiment. Day 618 timepoint represents the background fluorescence level before rehydration. The standard error of the mean was calculated and was too small to be observed on the graph. **(f)** Flow cytometry measurements of GFP+ and WT *B*. *frigoritolerans* A3E1 population fluorescence directly after separation from the soil on day 623 (bright green) and after overnight incubation in selective LB media (dark green). As a comparison, the fluorescence of a freshly inoculated *B*. *frigoritolerans* A3E1 population is presented (black).

Laboratory conditions often poorly reflect the stressful and varying field environments. The US Naval Research Laboratory (Washington DC) has a 12 m x 18 m x 14 m High Bay facility or “jungle room” designed to replicate a southeast Asian rainforest for system testing (Fig 4A). A variety of tropical plants native to Southeast Asian rain forests are maintained in the greenhouse, with an appropriate density of plants and foliage and a three-level canopy. It maintains live plant growth, a stream, pond, and rain is simulated via sprinklers operated for 5 minutes twice a week at 70 mm/hr. The temperature is held constant at 27°C with 80% humidity. We evaluated the ability of the GFP+ *B*. *frigoritolerans* A3E1 to maintain its function for long periods under these conditions.

The bacteria were mixed with soil in flowerpots (Methods). On April 12, 2019, two pairs of pots containing GFP+ and wildtype *B*. *frigoritolerans* A3E1 were prepared and placed in different locations. One pair was placed in the “jungle room” under a plant canopy (Fig 4A), and two additional pairs were placed in a storage room designed to mimic an enclosed man-made structure within a jungle environment, with 50–60% average humidity and a temperature of 21°C. In both locations, the pots were kept in ambient, non-sterile conditions and exposed to their environments.

The flowerpots in the jungle room were imaged periodically for 16 days between April 12 and April 26, 2019 (Fig 4B). Imaging was performed both under white light and fluorescent imaging conditions using a cell phone camera, a light filter (505 nm), and a hand-held blue-light LED (450 nm). The fluorescent signal from the pot containing the GFP+ bacteria was readily detectable for 10 days, after which it started to diminish, and mold contamination increasingly covered the surface of the pot (S6 Fig in S1 File). For these two pots, the experiment was halted on day 16 due to the mold contamination and low levels of fluorescence.

For the pots in the storage room, the experiment was continued. On November 4, 2020, 575 days after inoculation, the pots were imaged in a darkened room under a blue LED flashlight (450 nm). Low fluorescence could still be detected in the flowerpot with the GFP+ bacteria, and no signal was detected from the pots containing wildtype bacteria (S7 Fig in S1 File). Next, they were imaged in a fluorescent imager (Fig 4C) and fluorescent microscopy (S7 Fig in S1 File); fluorescence signal levels were low but still readily detectable without any intervention.

After 575 days, from one of the flowerpots, we incubated a small 20 mg sample of surface soil containing wild-type *B*. *frigoritolerans* A3E1 overnight in 3 mL LB media at 37°C and 250 rpm to outgrow bacteria for metagenomic sequencing. To assess the prevalence of new colonizing microbes in the soil sample, all resulting sequencer reads were aligned in an unsupervised manner to the Refseq genome database, and the number of hits for each isolate was countered. The results show the bacteria that have colonized the soil (S8 Fig in S1 File) chiefly among them are members of *Bacillus* (46%), *Pantoea (17%)*, and *Enterobacter* and *Escherichia* (9%) genera.

Since the pots dried during the months of storage without watering, we tested how hydration would impact the fluorescence signal. Watering was performed over five days, two times a day, spreading 5 mL of sterilized water onto each pot. The fluorescence at the surface of the soil with the GFP+ *B*. *frigoritolerans* A3E1 increased during this period (Fig 4D). The experiment was repeated after 618 days with the last pair of flowerpots to quantify bacterial growth by measuring the mean fluorescence of the pixels of the same area of the pot’s surface over time (Fig 4E). After this, on day 623, soil samples were taken from both the wildtype and GFP+ *B*. *frigoritolerans* A3E1 pots for flow cytometry measurements. The resulting cell populations were examined using flow cytometry. The soil-borne GFP+ *B*. *frigoritolerans* A3E1 population showed 5-fold lower median fluorescence than the original bacterial stock cultured in LB media (Fig 4F). However, when we streaked bacteria from the pots, picked individual colonies, and regrew them in LB media, the original and stored GFP+ strains showed similar fluorescence. This indicates that the ability to express GFP was only minimally impacted, if at all, despite being carried for almost two years.

### Long-term genetic stability

We sequenced the genomes of the wildtype and GFP+ *B*. *frigoritolerans* A3E1 strains before and after 575 days (S9 Fig in S1 File). Two isolates from the pots containing wildtype bacteria and one from the pot containing GFP+ bacteria were sequenced. The wildtype isolates each only picked up three mutations in the genome during this period, only two of which led to amino acid changes in genes (Table 2). One of these included an insertion in *yibJ* that occurs 23 amino acids from the stop codon of this 406 amino acid protein. This frameshift results in a frame without a downstream stop codon, likely leading to the disruption of the gene. Mutations in the *ylbJ* in *B*. *subtilis* are known to result in defective spore formation [53]. A replicated experiment in a separate flowerpot also picked up three mutations but at different positions (Table 2). Notably, a single gene cluster of ATP-dependent transporter genes had a mutation in the two different isolates of the wildtype pots, albeit in different genes in the cluster.

**Table 2:** Genomic mutations in *B*. *frigoritolerans* strains.

			*Bacillus frigoritolerans* ^a^					
Genomic locus^b^	Gene	Protein	Mutation	Location	A3E1	A3E1 after LTS^c^	GFP+ A3E1	GFP+ A3E1 after LTS^c^
*199*,*464*	*purR*	Pur operon repressor	C→T	I94	-	+	-	-
*444*,*240*	*nuoC*	NADH-quinone oxidoreductase subunit	G→C	A116	-	+	-	-
*444*,*331*	*nuoC*	NADH-quinone oxidoreductase subunit	C→T	A147S	-	+	-	-
*2*,*048*,*230*	*alaS*	Alanine tRNA ligase	T→C	H830P	-	+	-	-
*4*,*133*,*753*	*yibJ*	Sporulation integral membrane protein	+T	383^d^	-	+	-	-
*4*,*186*,*490*	*rnjA*	Ribonuclease J1	C→T	P43L	-	-	-	+
*4*,*286*,*301*	*ytrB*	ABC transporter ATP-binding protein	G→A	A103V	-	-	-	+
*4*,*292*,*517*	*bmrA*	ABC transporter ATP-binding protein / permease	A→G	G437	-	+	-	-
*4*,*480*,*722*	*hpr*	HTH-type transcriptional regulator	C→A	5’ UTR^e^	-	-	-	+
*4*,*586*,*517*	*baeS* ^f^	Signal transduction HAMP domain-containing histidine kinase	C→T	A34S	-	-	-	+
*5*,*435*,*792*	*immA* ^g^	ICE*Bs1* Immunity repressor	G->A	V12L	n/a	n/a	-	+
*5*,*435*,*788*			T->C	H14Q	n/a	n/a	-	+

^a^Each isolate is referred to as a concatenation of the isolation location and the sample name.

^b^Mutation’s genomic coordinates in the closest annotated reference species *Bacillus frigoritolerans* DSM8801.

^c^LTS: Long-Term Storage.

^d^Frameshift results in no stop codon in transcript.

^e^Gene name determined by homology search (reference genome annotation—A6755_RS21650).

^f^Mutation in RBS (16 bp upstream of start codon).

^g^Gene is part of the recombinant miniICE*Bs*1 cassette.

Similarly, the GFP+ strain picked up six mutations, only 2 of which were in the mini-ICE*Bs*1 region and none were within the payload (*gfp* expression cassette and *kan* resistance cassette). The two mutations within mini-ICE*Bs*1 were in *immA* (V12L and H14Q). This gene encodes a protease that functions as an inducer by degrading *immR*, a repressor of the *xis* promoter [54]. P_xis_ is the main promoter that controls the expression of the mini-ICE*Bs1* excision (*xis*), replication (*helP*, *nicK*), and conjugation (*conQ*) genes which could have deleterious effects on its host cell if expressed (Fig 2A). If these mutations decrease transcription or activity of *immA* that would result in better repression of the expression of the mini-ICE*Bs1* operon.

## Discussion

This work introduces a new species-indifferent paradigm for introducing a genetically encoded function into the environment. Typically, the carrier of the function is either a model organism, such as *E*. *coli* [4], or a single strain that is isolated from the environment for which engineering tools were laboriously developed [29, 55–59]. The broad species specificity of XPORT allows a payload to be pre-loaded into this strain and then transferred in parallel to many species isolated from the site-of-interest, from which the one that produces the best functionality, genetic stability, and growth. XPORT and automation allowed this project to proceed rapidly. The payload was inserted into the genomes of the isolated strains within three days of their receipt. Within a week, we had sequencing and functional data that allowed us to select the strain–a species we had not worked with before–to go on to the application testing phase. This approach requires that the isolate be culturable under lab conditions. However, it can go one step further, like recent *in-situ* approaches, where the transformation occurs in the environment in a species-targeted or untargeted way [11, 12, 40].

In this report, the “payload” is a very simple genetic device: just an expression cassette to achieve high expression levels of GFP. The constant expression of a reporter could be used for tag-and-track applications, where vehicle- and UAV- mounted GFP sensors have been developed that can scan for the presence of mud containing the manipulated bacteria [4, 60]. This mud could rub off on clothing or tires that move across the region. While simple, the device was still designed to operate in as wide a range of species as possible through the selection of genetic parts known to operate in diverse Gram+ species. The taggant could persist in soil for years or after a defined time or number of cell generations [61, 62].

More complex genetic devices could include sensors that respond to explosives, pollutants or soil nutrients, genetic circuits to implement signal processing, or pathways to produce chemicals or materials [18, 26, 63–65]. Design paradigms could be applied to increase the species range of these devices [66–69], which could then be pre-loaded into XPORT as a means to rapidly transfer the functionality to many of the species in an environmental sample in parallel. Obtaining reliable function across species could be achieved through “universal parts,” designed to function across species, feedback loops to self-adjust to cell volume, cytoplasmic conditions, or resource availability. Similarly, transporting the molecular machines required by the central dogma could be used to create a “virtual machine” within the recipient cell [70]. Collectively, these approaches could lead to genetic devices that function reliably when inserted into the genome of a new species.

Engineered bacteria used for field applications are needed to advance environmental biotechnology beyond its lab-demonstration nascency. It is particularly important for bioremediation [71–73], environmental biosensing [74, 75], agriculture [18, 76], object provenance and living materials [22, 33], microbiome engineering [77–79], and climate change and sustainability solutions [80–82]. *B*. *frigoritolerans* is the isolate that emerged from the screen as it generated high GFP expression, rapid growth in the lab, and maintained a high carrying capacity in soil in laboratory experiments. This strain maintained high levels of GFP expression for nearly two years in a dry storage area. We were surprised that this did not lead to extensive mutations to the genome and to the payload device. We have observed in other systems that high levels of recombinant gene expression lead to rapid evolutionary breaking of the genetic device and secondary mutations to the genome to mitigate its impact [35, 65, 83, 84], likely due to its draw on resources, particularly ribosomes [85]. It is likely that the long-term persistence of *B*. *frigoritolerans* is enabled through spore formation, initiated in the drier environment, and then allowing spore to be maintained in the soil for long times and then re-accessed by germination when rehydrated [86]. Such a mechanism could be further utilized for in-field applications as bacterial spores, instead of geminated cells, could be generated and deployed to the field and activated by rehydration [22].

There are potential advantages to introducing a desired genetic function to a native isolate from the environment. For one, it is naturally adapted to that environment and may have the needed stress responses for long-term persistence. It may also contain resistance mechanisms to other bacterial species, phage, protozoa, and antifungals [87–89]. Indeed, we observed rapid overgrowth of the bacteria by fungi when inoculated in the jungle room, which is wetter than the environment from which they were isolated. Second, it reduces the likelihood of negatively impacting the environment, including microbiome dysbiosis, which may simplify regulatory approval. The genetic payload would be the only new DNA introduced to the environment, which could be targeted by degradation machinery [61], thus reverting the isolate to what was there originally. The entire organism could be limited in its persistence by physically isolating it through encapsulation [4, 90], auxotrophy [91–93], or the incorporation of genetically encoded “safety switches” [94, 95].

In the jungle room, it is interesting that fungi initially bloomed and interfered with the bacterial growth. The jungle room is a very different environment than that from which the bacteria were isolated and presumably, the fungal species are different. One approach to this problem would be to engineer the bacteria to produce antifungals that interfere with the species in the target environment [13]. Phenazine biosynthesis pathways, for example, can be engineered into bacteria [96] and confer resistance against fungi [97]. A similar problem could be cause by local phage that disrupt bacterial growth. Bacteria from the same environment could contain immunity (CRISPR arrays) against the local phage, but these are absent from bacteria that have not been similarly exposed. This could be addressed by engineering the CRISPR arrays to have sequences that match phage from the environment-of-interest.

We see multiple advantages for approaches using environmentally native chassis for fieldable applications. First, it can mitigate adverse environmental impact risks from introducing foreign, potentially invasive species [98]. Furthermore, it will not add foreign genetic information that can survive long after the death of the deployed bacteria [5, 99], traits, or species to the microcosm other than the recombinant payload. Second, screening the vast bacterial diversity of the target microbiome against one genetic design reduces the need for host-specific optimization of the genetic circuit. Third, native bacteria can maintain in-field persistence and performance over more extended periods than non-native model bacteria [100, 101].

## Materials and methods

### Strains, media, plasmids, and chemicals

*E*. *coli* NEB 10-beta (New England BioLabs (NEB), Ipswich, MA, #C3019I) was used for cloning. Kanamycin (5 μg/mL, GoldBio, St Louis, MO, #K-120-5) was used for liquid and solid media selection. DNA oligos and genes were ordered from Integrated DNA Technologies and Twist Biosciences. *B*. *subtilis* XPORT (JH642, trp-phe- ICEBs1+) [38, 40] was used as the parent donor conjugation strain. *B*. *subtilis* XPORT were grown in LB media (Becton Dickinson (BD), Franklin Lakes, NJ, #244630), and 1.5% w/v agar plates (BD #247940). The P15a plasmid used to transform the GFP payload into the mini-ICE*Bs*1 cassette of *B*. *subtilis* XPORT was based on pICEGFP05, described in Meng et al. [38] (S10 Fig in S1 File). Genetic part sequences are provided in S2 Table in S1 File. Plasmid DNA was introduced into *B*. *subtilis* XPORT through natural transformation. D-alanine (100 μg/mL) (Sigma, St. Louis, MO, A7377) was added to the LB media or MD media for counter selections. Tris-Spizzizen Salts (TSS) media was used for ICE conjugations on plates (per L: 2 g NH_4_Cl (Fischer A649), 0.35 g K_2_HPO_4_∙3H_2_O (VWR #0705), 6 g Tris base (Affymetrix, Santa Clara, CA, 75825); pH to 7.5 with HCl (EMD, Millipore, Burlington, MA, HX0603-3)). Mating plates were made with TSS, 125 mM MgCl_2_ (Sigma 230391) and 1.5% w/v agar (BD 214010).

### Soil sampling

To avoid contamination, soil samples were collected using a covered collection trowel while changing sterile nylon trowel covers between samples. Each sample was collected from several locations around the same area to avoid local abnormalities and mixed to fill one 50 mL tube (Thermo Fischer, Waltham, MA, Corning 4558), the tube was tightly capped. Surface samples were collected directly from the ground. Samples from the 2m deep human-build structure in site A were taken using a drilling machine that excavated the soil from the 2m depth to a surface pile. From there, it was collected using the trowel. Soil samples from site A and site D were stored at 4°C and room temperature for 11 days and 1 day, respectively, until plated on LB agar plates and incubated overnight at 37°C. The “in culture” sample preparation (method 3) was done four months after the collection of samples. Soil sampling permits were granted by the Israeli ministry of defense, precise coordinates of sampling are unavailable, but the area is described in the results section.

### Collection of recipient cells for conjugation

To resuspend bacteria from the soil samples (except for sample D3, which was a liquid sample), 5g of soil from each sample were mixed with 10 mL of DW autoclaved water and were incubated for one hour at 37°C and 250 rpm (Multitron Pro 2, INFORS HT, Weymouth, MA, INF-68798). Liquid sample D3 was also directly incubated for one hour at 37°C and 250 rpm. Soil particles were pelleted by spinning 100 g, 1 min, RT (Eppendorf, Hamburg, Germany, 5910R). The bacteria-containing supernatant was collected. Next, three methods were used to prepare recipient isolates for conjugation (S1 Fig in S1 File).

Method 1 (“in culture”): All samples were incubated at 37°C and 250 rpm for an additional overnight. The next morning, 2 mL of the bacteria-containing water from samples D3 and D4 were saved for mating.

Method 2 (“colony pooling”): 1 mL of bacteria-containing water was used from all samples (except sample A1) to inoculate 50 mL LB in 250 mL Erlenmeyer flasks (Corning, 4450–250) and overnight incubated at 37°C, 250 rpm (New Brunswick Scientific, Enfield, CT, Innova 44). After incubation, 10 ul of the overnight cultures were used to plate an LB agar plate and were incubated overnight at 37°C. All the colonies on each plate (10^2^–10^3^) were collected by resuspension in LB media resulting in a pooled bacteria resuspension. The pooled mixture was incubated overnight in 250ml Erlenmeyer flasks containing 50 mL of LB at 37°C and 250 rpm. The resulting cultures were saved for conjugation.

Method 3 (“isolated colony”): 1 mL of bacteria-containing water from samples A1 and A3 were used to inoculate 50 mL LB in 250ml Erlenmeyer flasks and incubated overnight at 37°C, 250 rpm. One single colony from each sample was picked and used to inoculate 3 mL of LB in 14 mL culture tubes (Thermo-Fisher #352059) that were incubated overnight at 37°C and 250 rpm. The resulting cultures were saved for conjugation. We note that lower temperatures for incubations may also be used and be more appropriate for the growth and diversity of soil bacteria obtained.

### Conjugation using B. subtilis XPORT

The conjugation donor, *B*. *subtilis* XPORT with the mini-ICE*Bs*1 cassette carrying the payload DNA, was streaked from a glycerol stock onto LB agar plates with D-alanine overnight at 37°C. One isolated colony of the donor was used to inoculate 2 mL of LB in 14 mL culture tubes. The recipient cells from each sample were inoculated using 1 mL of the resulting culture from each method in 2 mL of fresh LB in 14 mL culture tubes. The cultures were incubated for 3 h at 37°C and 250 rpm. Next, the cultures were diluted 1:40 into 10 mL of LB. These dilutions were made into 250 mL Erlenmeyer flasks and shaken at 250 rpm and 37°C for 1 h, after which 1 mM of IPTG was added to the donor cell culture to induce the ICE conjugation genes, and the cultures were allowed to continue incubation for an additional hour under the same conditions. Donor-recipient conjugations for all samples were performed using filter mating on TSS agar as previously described [38, 40]. Briefly, 100 μL of Donor and Recipient cells were mixed and co-cultured on filters placed on TSS agar plates for 3 h at 37°C. After this incubation, the co-cultures were streaked on LB selective agar plates supplemented with kanamycin and were put into overnight incubation at 37°C. The plates did not have D-alanine to select against the XPORT donor. The next day, plates were placed on a Blue Light Transilluminator (Thermo-Fisher), and 25 fluorescent colonies were picked to inoculate 3 mL of LB in 14 mL culture tubes supplemented with kanamycin and incubated overnight at 250 rpm and 37°C. The resulting cultures of conjugated isolates were used for downstream assays and to make bacterial glycerol stocks (20% v/v; VWR BDH1172-1LP).

### Flow cytometry analysis

Cytometry was performed using a BD LSR Fortessa flow cytometer with the HTS attachment (BD). Samples were prepared by aliquoting 10 μL cell culture into 250 μL PBS. All samples were run in standard mode at a flow rate of 2 μL/s. The fluorescence of events was measured using the FITC (495 nm) laser and the FITC channel (PMT voltage of 400 V). The FSC and SSC voltages were 550 V and 225 V, respectively. At least 50,000 events were collected for each sample, and the Flowjo V10.7.1 software was used for analysis. The FITC median was calculated for the distributions (S1 Table in S1 File). The gating strategy was to include all events of bacterial cells between SSC-A 10^3^−10^5^, FSC-A 5 × 10^2^ − 10^5^ and SSC-H 10^3^−10^5^, FSC-H 5 × 10^2^ − 10^5^.

### ICE-cassette genomic localization of GFP+ soil bacteria

To identify the location of the payload insertion, we processed the next-generation sequencing data of GFP+ strains in the following way. First, all genome assemblies were aligned using BLASTn against the mini-ICE*Bs1* sequence. Second, each relevant contig that was found to harbor the miniICE*Bs1* cassette has been compared to the closest reference using BLAST to find the most probable genomic insertion coordinates. Based on the reference annotation, we determined whether the insertion occurred at the tRNA-Leu gene. Genomic coordinates of the mini-ICE*Bs1* in all isolated strains are detailed in S1 Table in S1 File. Next-generation sequencing methods and the analysis tools used are described in the supplementary methods section.

### Growth rate measurements

Bacteria were streaked from bacterial glycerol stocks onto LB agar plates containing kanamycin. Colonies were picked and incubated overnight at 37°C and 900 rpm in a deep 96-well plate (USA Scientific #1896–2000). A 5 μL aliquot of the culture was inoculated into 195 μL of fresh LB media with kanamycin in a 96-well plate (Thermo Scientific Nunc #137101) that was incubated for 18 h at 37°C and 900 rpm, 3 mm shaking amplitude (INFORS-HT Multitron Pro). OD (600nm) was measured every 15 minutes. The OD_600_ was plotted on a log_2_ axis, and doubling time was calculated as the slope of the best-fit line.

### Functional stability over time in sterile and non-sterile soil

All 25 engineered strains were streaked on LB agar plates from glycerol stocks. Single colonies were picked into 200 μl LB in 14 ml culture tubes and incubated at 37°C and 250 rpm until their OD_600_ reached 1 by measuring it in a shaking plate reader (Spark, Tecan). Each culture was transferred into 5 ml fresh LB media in 50 ml tubes and incubated overnight at 37°C and 250 rpm. 1 gram of sterile or non-sterile soil was added to each culture and mixed together for another 2 hours. The mixtures of cells and soil were harvested by centrifuging 4800g for 10 minutes (Multifuge X3R, Thermo scientific) at RT. After centrifugation, the cells and soil were thoroughly mixed with 1 ml 1×PBS (Hylabs, Rehovot, Israel, BP507/500D) and moved into 96 flat bottom well plates (NUNC, Thermo Scientific, 260836) in quadruplicate 300μl each. The plates were incubated at 24°C with 90% humidity (Multitron Pro 2, INFORS). Fluorescence of the GFP (excitation 485; emission 508) was measured in a plate reader (Spark, Tecan, Männedorf, Switzerland) every day for 10 days and then every other day. The measurements were taken in different gains (100, 110, 120, 130, 140, 150) with a total measurement time of 4 minutes for each plate. After the four repeats were measured, the plates were put back in the incubator. For each time point, the average fluorescence of gain of 100 was calculated as well as normalized fluorescence that was calculated by setting for each strain an initial fluorescence to 1, extrapolating the corresponding gain from a calibration curve plotted according to the measurement in the different gains (exponential curve) and using this gain for all the later time points.

### Soil flowerpot experiments

Sterile soil was made by taking silt loam soil from Cambridge, MA, and autoclaving it in a liquid cycle for 30 minutes at 121°C (CSS Autoclave, ADV_PB, Billerica, MA). Wildtype and GFP+ *B*. *frigoritolerans* A3E1 strains were streaked onto LB agar plates with kanamycin. Single colonies were picked into 4 mL LB media in 14 mL culture tubes and incubated at 37°C and 250 rpm. When the OD_600_ reached 1, one mL of each culture was transferred to 1 L fresh LB media in 2.8L Erlenmeyer flasks (Corning, 4420-2XL) and incubated overnight at 37°C and 250 rpm. The next day, 5 g of sterile soil was added and cultured together for another 2 hours under the same conditions. The mixture of cells and soil was harvested by centrifuging at 5,000 g for 10 minutes (Thermo Sorval RC 6+). After centrifugation, the cells and soil were thoroughly mixed and moved into 2-inch diameter flowerpots (Shop Succulents, 2" plastic pots, B073VPZD33, Amazon, Seattle, WA). Each flowerpot was filled with soil-bacteria pellets from about 4L of culture, which corresponds to about 20 g (dry weight) of soil. The flowerpots were put into a secondary containment plastic tray (Nalgene, WVR, West Chester, PA, 62662–875). The soil was imaged using a blue LED flashlight (450 nm, 200 lumens; WAYLLSHINE Scalable Blue LED, Amazon) and through Longpass 505 nm filter (Chroma AT505DC, Bellows Falls, VT). Soil rehydration of the flowerpots was done twice a day for five consecutive days at around noon and 6 pm, spreading 5 mL of MiliQ water equality on the soil surface. The quantification of fluorescence for the rehydrated flowerpots was performed 1 minute after the addition of water at noon, using a Chemidoc MP gel imager (Bio-rad, Hercules, CA), under Blue EPI illumination and through 530/28 nm filter, with an exposure time of 1 s.

### Tropical high bay “jungle room” experiment

On April 11, 2019, Soil pots containing GFP+ and WT *B*. *frigoritolerans* A3E1 were shipped at ambient conditions covered with parafilm wrappings to the high-bay facility of the Naval research laboratory for autonomous systems research, Washington DC. The soil pots were placed in a leak-proof secondary containment tray (The Lab Depot, Dawsonville, GA, 107334) inside the jungle room on arrival on April 12, 2019. They were placed beneath a *Yellow poinciana* tree and next to a small stream. The flowerpots were exposed to their surroundings, but the tree canopy reduced the impact and mechanical disruption from raindrops. The flowerpots were imaged irregularly every few days throughout the experiment by taking the soil samples to the indoor facility and using fluorescent measurements using an iPhone SE 12.0-megapixel digital camera under white light illumination or in a dark room with a blue LED flashlight (450 nm, 200 lumens) and through longpass 505 nm filter. After imaging, the soil pots were returned immediately to the same location in the jungle room. The long-term storage experiment was conducted in parallel with identical soil pots that were stored in a lab storage space, exposed to their surroundings and under ambient conditions. The long-term stored pots lay undisturbed until imaged before rehydration 575 days after the experiment had started. Permit for the experiment was granted by the Naval research laboratory.

### Separation of bacteria from the soil for flow cytometry

From bacteria-infused soil flowerpots, 20 mg of surface soil was collected and put in microcentrifuge tubes. A 200 μL aliquot of PBS buffer was added to each sample that was vigorously vortexed for 10 min. Soil sedimentation was performed by centrifugation at 1000 g, RT for 2 min (Eppendorf 5424R) to separate bacteria from larger soil particles. The supernatant was analyzed using flow cytometry.

### Bacteria infused soil textile smearing

GFP+ and wildtype *B*. *frigoritolerans* A3E1 cultures were separately mixed with soil as detailed above for the flowerpot experiment. The resulting bacteria-infused mud was spread evenly on a textile similar to those used by the US army military (camouflage nylon cotton ripstop fabric) worn by a volunteer. The bacteria had to be periodically sprayed with MiliQ water (Millipore, Advantage A10), as the fluorescence signal was significantly increased when the bacteria-infused soil was wet. Imaging was done using a Google Pixel 2 cell-phone camera under white light or illumination by a blue LED flashlight and through amber protection goggles longpass 505 nm filter.

The signal stability experiment was done with similarly bacteria-infused soil spread on a uniform and left in ambient conditions of the lab on a clothes hanger throughout the experiment. Each day at noon, the smeared textile was lightly sprayed with MiliQ water, and within 1 minute, it was imaged with iPhone 11 camera through amber UV protection goggles (Invitrogen, Waltham, MA, Safe Imager, 287.1) illuminated by the blue LED flashlight and Chemidoc MP gel imager (Bio-rad), under Blue EPI illumination and through 530/28 nm filter, with an exposure time of 0.15 s. Soil clumps (i.e., ‘breadcrumbs’) were generated by covering textile with 10 g of *B*. *frigoritolerans* GFP+ infused soil mixture that was smeared on the textile and allowed to dry for two days. After drying, the textile was gently hit with a spatula to allow for soil clumps to fall to the ground.

Laundry experiments were done using a twin tub washing machine (Zeny, 1669A, Amazon) and a uniform shirt (camouflage nylon cotton ripstop fabric) smeared with 10 g of *B*. *frigoritolerans* A3E1 GFP+ infused soil mixture. Washing cycles were done for 10 minutes with 10 L of 20°C or 60°C of tap water. Detergent (XTRA, Church and Dwight Co., LDLBB-LN1-05) was used with a concentration of 1 mL / 1 L of water. After the washing cycle, the textiles were moved to the spin-dry tub and spun for 5 minutes to remove residual detergent and water. After which areas around the stain which used to contain the bacteria-infused soil smear were ‘imprinted’ by pressing an LB agar plate supplemented kanamycin for 10s against the fabric and incubated overnight at 37°C. The washing machine wastewater was collected from the washing machine and used for monitoring experiments. For each washing experiment, 10 μL unconcentrated and 2 mL of wastewater concentrated to 100 μL by spinning for 3 minutes at 10,000 g and RT (Eppendorf, 5424R) and resuspending pellet in LB, were streaked on LB agar plate supplemented with kanamycin and incubated overnight at 37°C. Colony-forming units (CFU) of fluorescent colonies only in triplicate plates were counted to yield the CFU results presented. Colony fluorescent imaging was done using a Chemidoc gel imager. B. frigoritolerans A3E1 GFP+ biofilm regrowth was done with textile after washing with cold water (see above) by covering two 10 X 5 cm areas of the shirt with 250 mL of LB agar supplemented with kanamycin. After the agar solidified, the shirt was moved into a plastic tray (Nalgene, WVR, 62662–875), covered with a plastic sheet to reduce evaporation, and incubated for 48h at 37°C. After incubation, the remaining agar was removed, and biofilms were readily detectable on the textile using blue light and safe-stain goggles (see above) and by imaging in a Chemidoc gel imager.

## Supporting information

S1 File Supporting information file. Includes figures S1-S10, tables S1-S3, and supporting methods.
(PDF)

S2 File Family-level 16S analysis abundance analysis data for soil samples. (CSV)
