# Supplementary material for: Parallel engineering of environmental bacteria and performance over years under jungle-simulated conditions
Source: PLoS One. 2022 Dec 14;17(12):e0278471. doi: 10.1371/journal.pone.0278471 (PMC9750038; doi:10.1371/journal.pone.0278471)
Supplement: S1 File — Includes figures S1-S10, tables S1-S3, and supporting methods. (PDF) [file pone.0278471.s001.pdf]

Supporting Information for:

Parallel engineering of environmental bacteria and performance over years under jungle-simulated conditions

Yonatan Chemla[1¶], Yuval Dorfan[1¶], Adi Yannai[2¶], Dechuan Meng[1], Paul Cao[3], Sarah Glaven[4], D. Benjamin Gordon[1,3], Johann Elbaz[2], and Christopher A. Voigt[1,3*]

[1] Synthetic Biology Center, Department of Biological Engineering, Massachusetts Institute of Technology, Cambridge, Massachusetts, United States.

[2] School of Molecular Cell Biology & Biotechnology, Faculty of Life Science, Tel Aviv University, Tel Aviv, Israel

[3] The Broad Institute of MIT and Harvard, Cambridge, Massachusetts, United States

[4] Center for Bio/Molecular Science and Engineering, Naval Research Laboratory, Washington DC, USA

¶ These authors contributed equally to this work.

* Corresponding author

E-mail: cavoigt@gmail.com

Supporting Figures **2**

S1 Fig. Environmental samples and preparation methods of recipient isolates for mini-ICEbs1 mating. *2*

S2 Fig. Genome assembly of bacteria, distance, and differences to their closest references. *5*

S3 Fig. Performance of individual engineered isolates in soil *6*

S4 Fig. Traits and relativeness of B. frigoritolerans to engineered strains. *7*

S5 Fig. Quantification of engineered isolates in environmental samples. *8*

S6 Fig. Image of bacteria-infused soil during simulated environment experiment. *9*

S7 Fig. Imaging of long-storage B. frigotolerans A3E1 bacteria-infused soil before and after rehydration *10*

S8 Fig. Metagenomic abundance of bacteria that colonized the soil throughout the long storage. *12*

S9 Fig. Comparing genomic assembly before and after long-¬¬term soil incubation. *11*

S10 Fig. Payload plasmid map. *13*

Supporting Methods **14**

Supporting Tables **14**

S1 Table. Undomesticated soil bacteria isolated in this study. *18*

S2 Table. Genetic part sequences. *19*

S3 Table. 16S sequences of isolated undomesticated soil bacteria. *19*

Supporting Figures

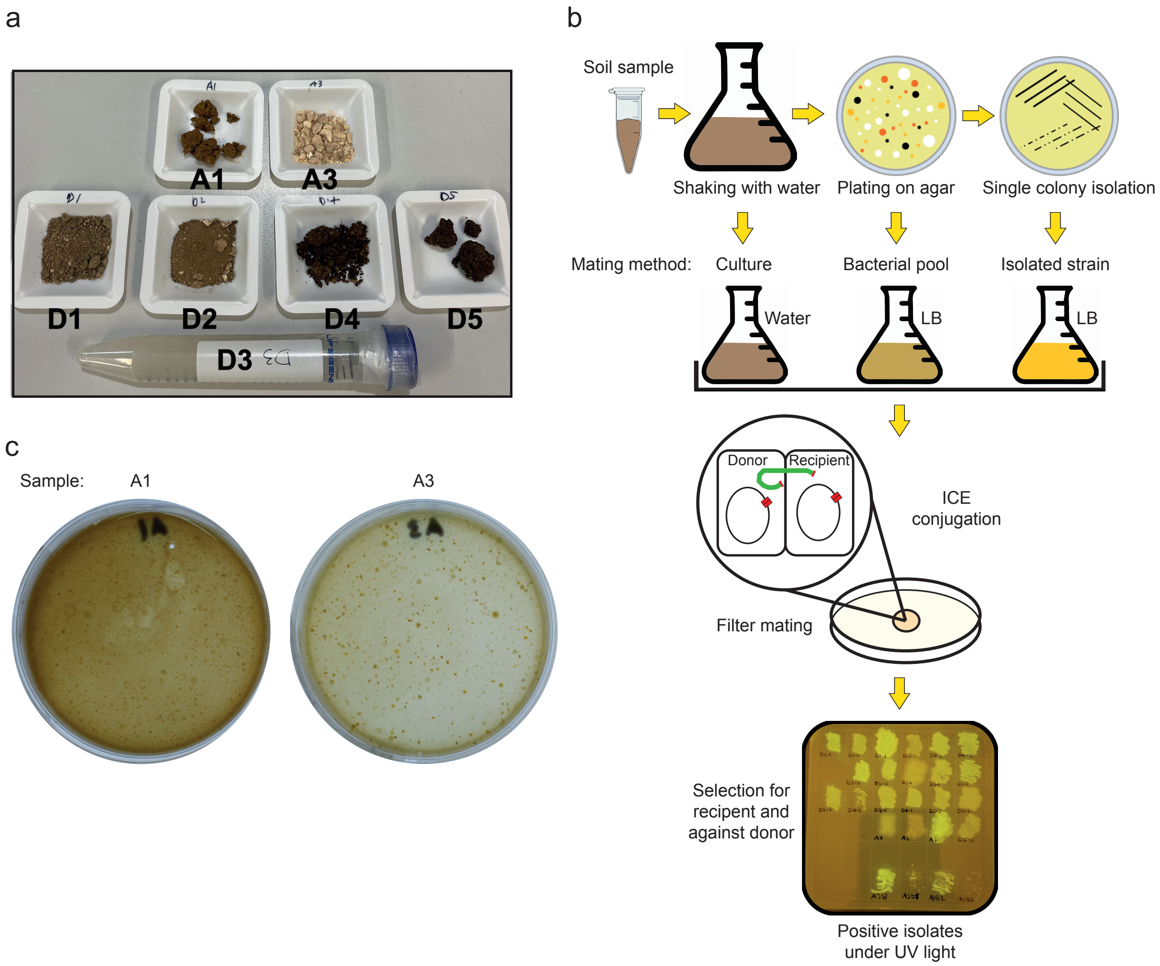

S1 Fig. Environmental samples and preparation methods of recipient isolates for mini-ICE*bs*1 mating. **(a)** Image of the soil and surface water samples used in the study **(b)** Scheme of the three isolation methods used to prepare recipient strains for mating, ICE conjugation of recipient and donor strains, and image of successfully engineered undomesticated isolates, expressing GFP, on a selective LB agar plate, imaged under UV light. **(c)** Representative agar plates showing multi-species colonies cultured from two soil samples: A1 and A3, before isolation and mating.

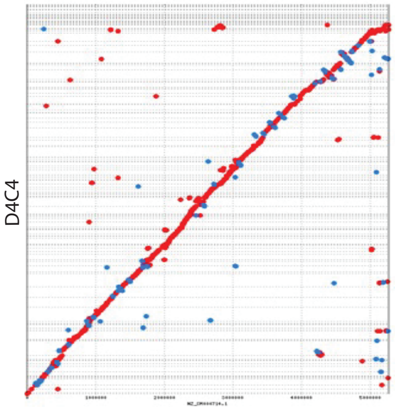

B. cereus

Refrence | Sample
Total Length (bp) | 5,269,725 | 5,468,156
Pct Covered | 79.26 | 76.35
Pct Identity | 91.06 | 91.06

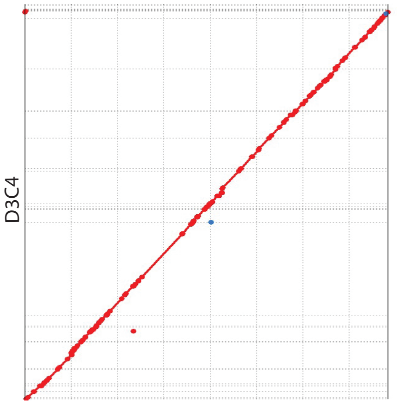

B. amyloliquefaciens

Total Length (bp) | 3,918,589 | 3,996,116
Pct Covered | 93.80 | 91.65
Pct Identity | 98.38 | 98.38

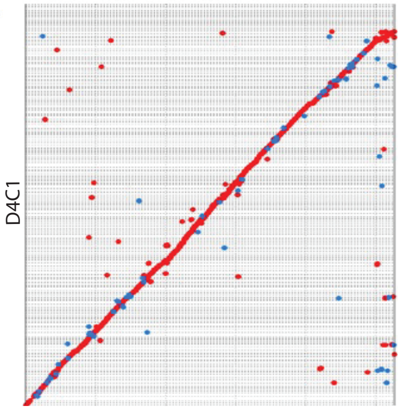

B. cereus

Total Length (bp) | 5,269,725 | 5,486,430
Pct Covered | 79.14 | 76.15
Pct Identity | 91.08 | 91.08

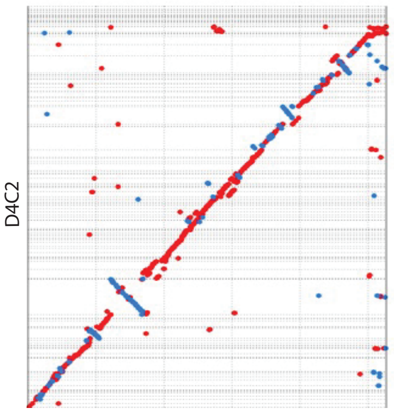

B. cereus

Total Length (bp) | 5,269,725 | 5,464,717
Pct Covered | 79.27 | 76.38
Pct Identity | 91.07 | 91.07

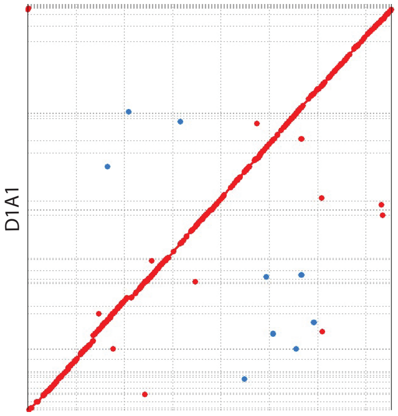

B. pumilus

Total Length (bp) | 3,763,493 | 4,226,344
Pct Covered | 87.10 | 77.53
Pct Identity | 88.61 | 88.61

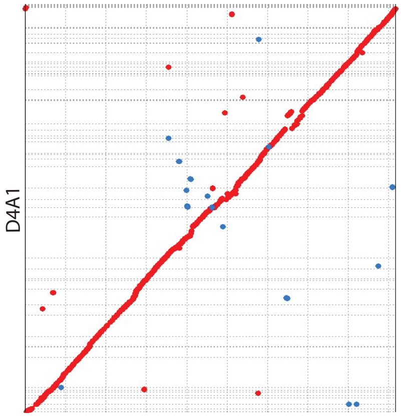

B. lysinibacillus sp. YS11

Total Length (bp) | 4,584,915 | 4,999,182
Pct Covered | 75.26 | 69.07
Pct Identity | 88.37 | 88.37

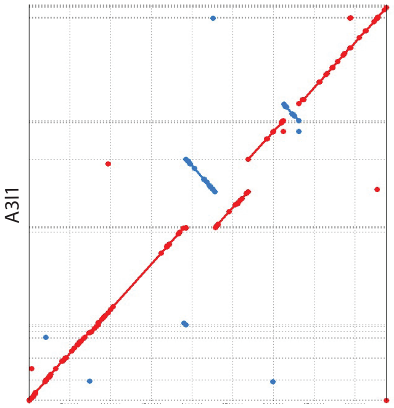

B. paralicheniformis

Total Length (bp) | 4,389,210 | 4,761,599
Pct Covered | 95.53 | 87.88
Pct Identity | 99.20 | 99.20

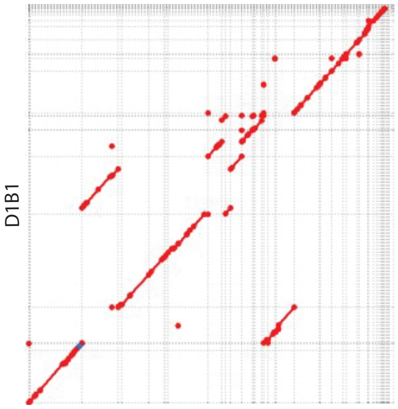

B. velezensis

Total Length (bp) | 4,134,697 | 4,489,721
Pct Covered | 91.01 | 83.83
Pct Identity | 99.19 | 99.19

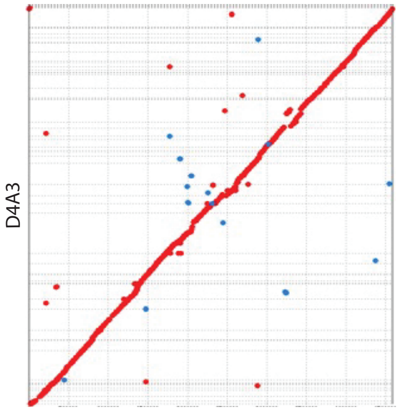

B. lysinibacillus sp. YS11

Total Length (bp) | 4,584,915 | 5,001,116
Pct Covered | 75.38 | 69.42
Pct Identity | 88.36 | 88.36

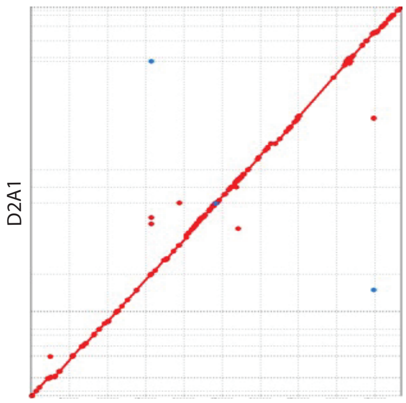

B. lysinibacillus fusiformis

Refrence | Sample
Total Length (bp) | 4,843,789 | 4,741,167
Pct Covered | 88.81 | 90.52
Pct Identity | 98.82 | 98.82

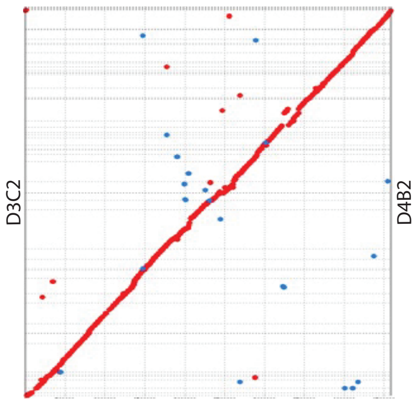

B. lysinibacillus sp. YS11

Total Length (bp) | 4,584,915 | 4,719,696
Pct Covered | 75.47 | 73.40
Pct Identity | 88.39 | 88.39

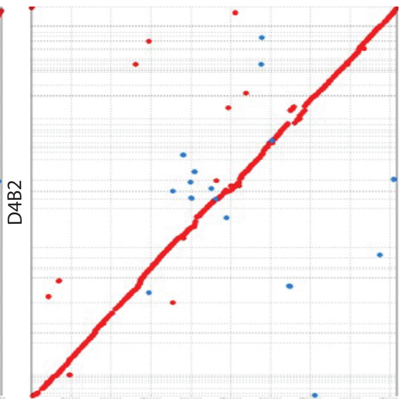

B. lysinibacillus sp. YS11

Total Length (bp) | 4,584,915 | 4,707,442
Pct Covered | 75.30 | 73.46
Pct Identity | 88.37 | 88.37

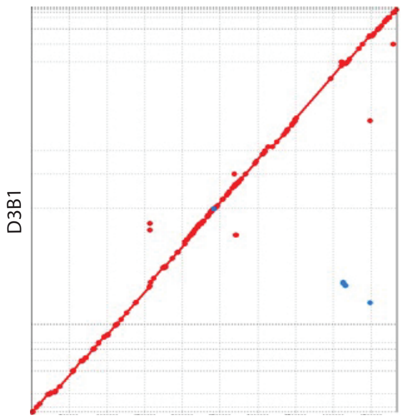

B. lysinibacillus fusiformis

Total Length (bp) | 4,843,789 | 4,841,261
Pct Covered | 88.83 | 88.69
Pct Identity | 98.81 | 98.81

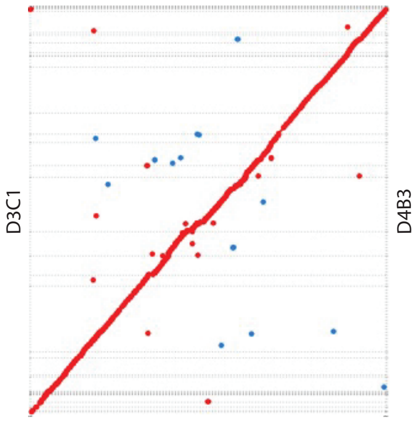

B. cereus

Total Length (bp) | 5,427,083 | 5,652,454
Pct Covered | 80.15 | 76.63
Pct Identity | 91.93 | 91.93

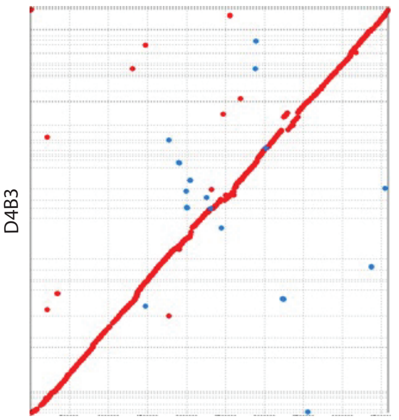

B. lysinibacillus sp. YS11

Total Length (bp) | 4,584,915 | 4,983,765
Pct Covered | 75.24 | 69.28
Pct Identity | 88.37 | 88.37

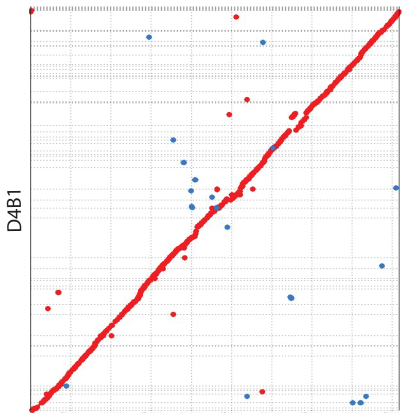

B. lysinibacillus sp. YS11

Total Length (bp) | 4,584,915 | 5,059,386
Pct Covered | 75.48 | 68.75
Pct Identity | 88.38 | 88.38

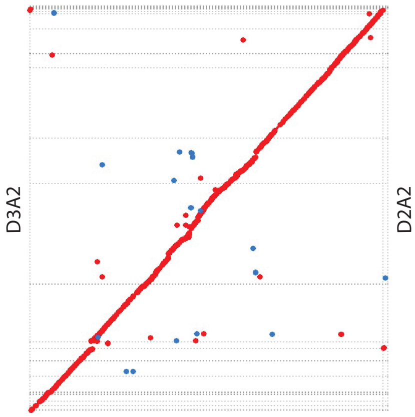

B. thuringiensis

Total Length (bp) | 5,314,794 | 5,565,417
PPct Covered | 81.10 | 77.50
Pct Identity | 91.66 | 91.66

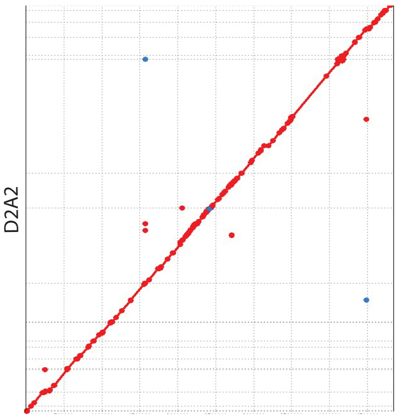

B. lysinibacillus fusiformis

Total Length (bp) | 4,843,789 | 4,815,192
Pct Covered | 88.69 | 89.09
Pct Identity | 98.81 | 98.81

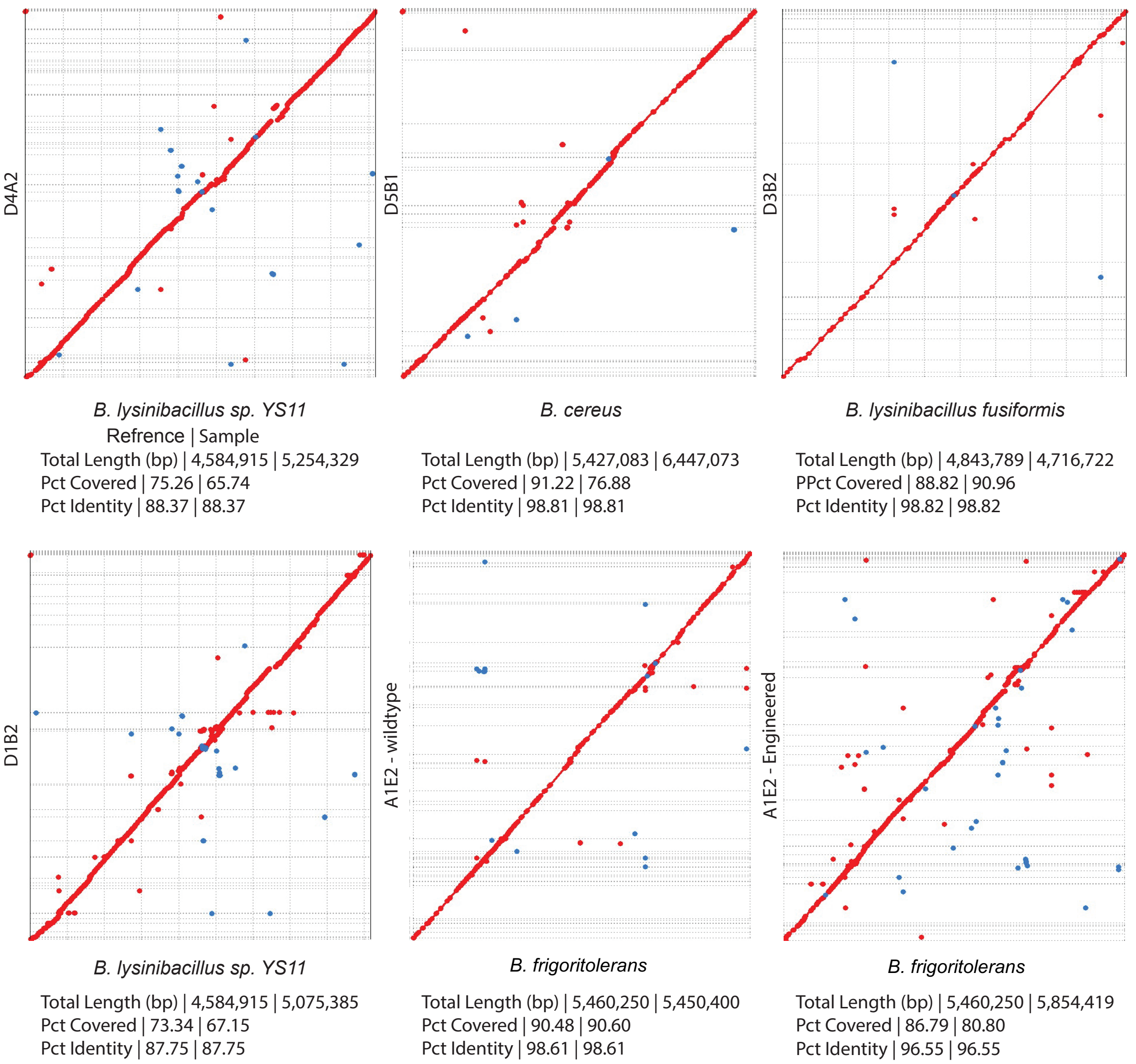

S2 Fig. Genome assembly of bacteria, distance, and differences to their closest references. MUMmer plots present the undomesticated bacteria shotgun genome assembly on the Y-axis compared with their closest NCBI reference on the X-axis. Each horizontal and vertical lines represent one assembly contig of the novel assembly and the reference, respectively. Below each plot is the total length of the assembly, the reciprocal percentages of coverage and identity of the reference genome (left), and the novel assembly (right).

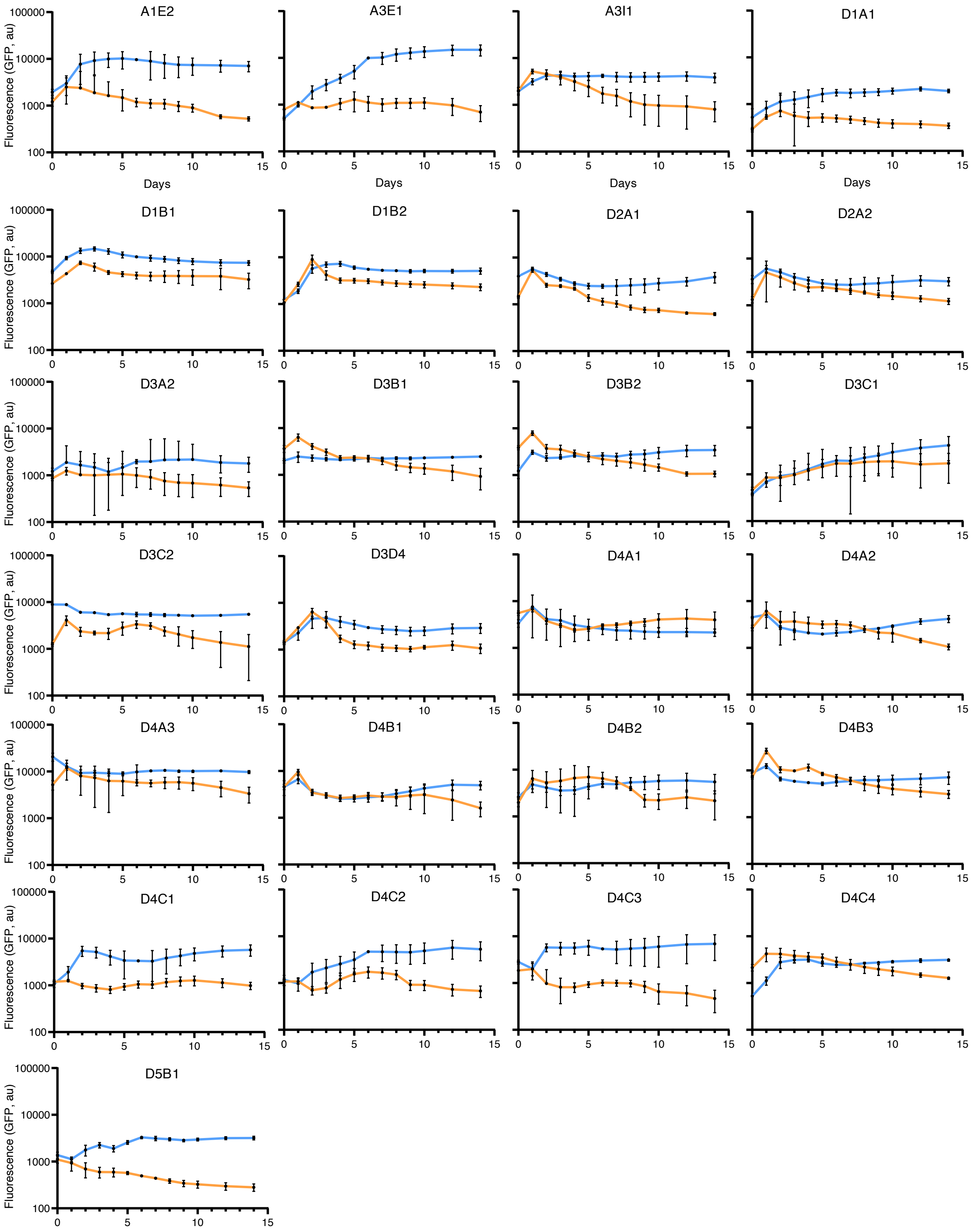

S3 Fig. Performance of individual engineered isolates in soil. Individual engineered bacterial isolates' total function over time in soil, measured by GFP fluorescence when reintroduced into sterile (blue) and nonsterile (orange) soil from site D. Error bars represent standard deviation.

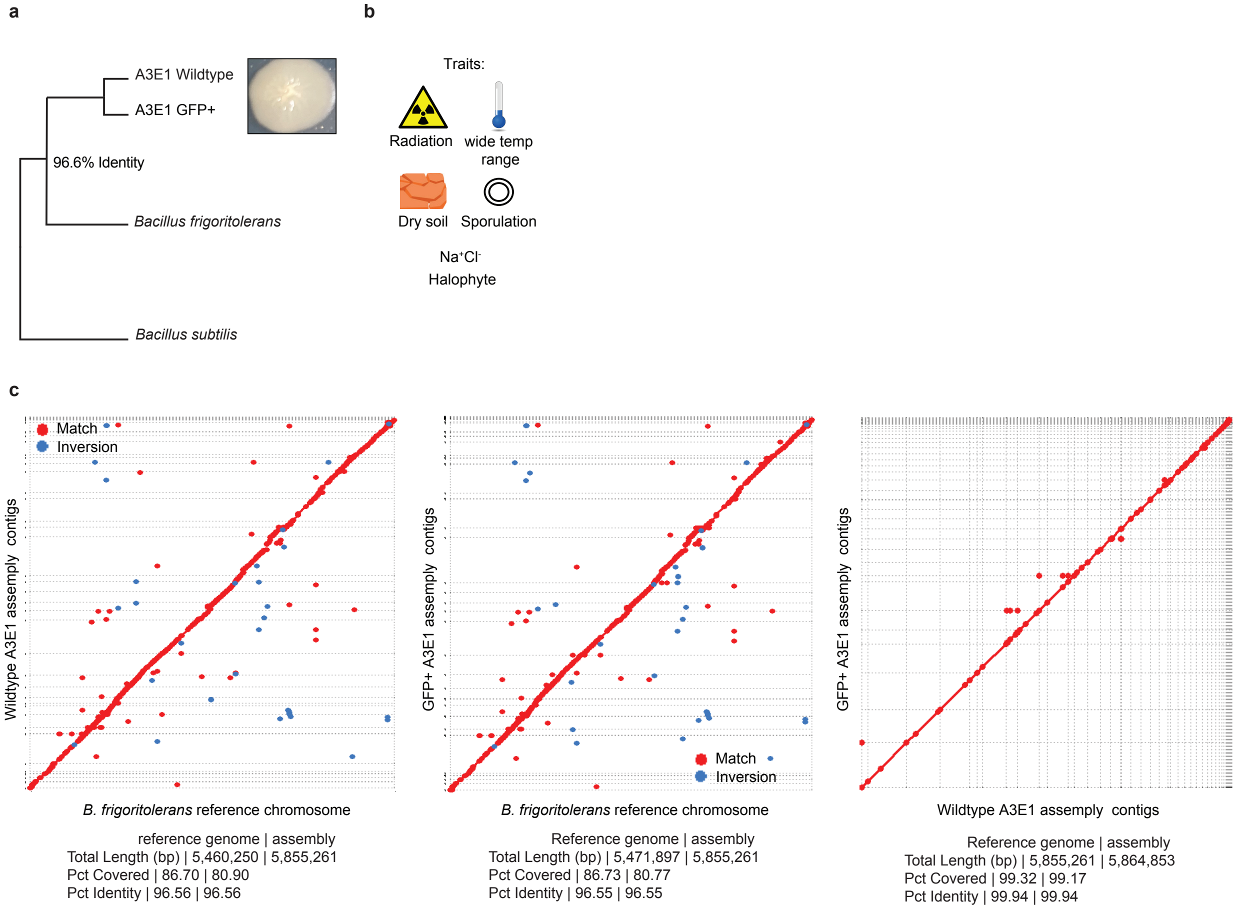

S4 Fig. Traits and relativeness of *B. frigoritolerans* to engineered strains. (a) Phylogeny of isolated strains compared to their closest annotated reference – *B. frigoritolerans*. **(b)** Tolerance traits of *B. frigoritolerans* documented in the literature[1,2] **(c)** MUMmer plots comparing the genomic assembly of wildtype (left) and GFP+ (middle) *B. frigoritolerans A3E1* to its closest NCBI reference – *B. frigoritolerans*. And a genomic assembly comparison between wildtype and GFP+ *B. frigoritolerans A3E1* (right).

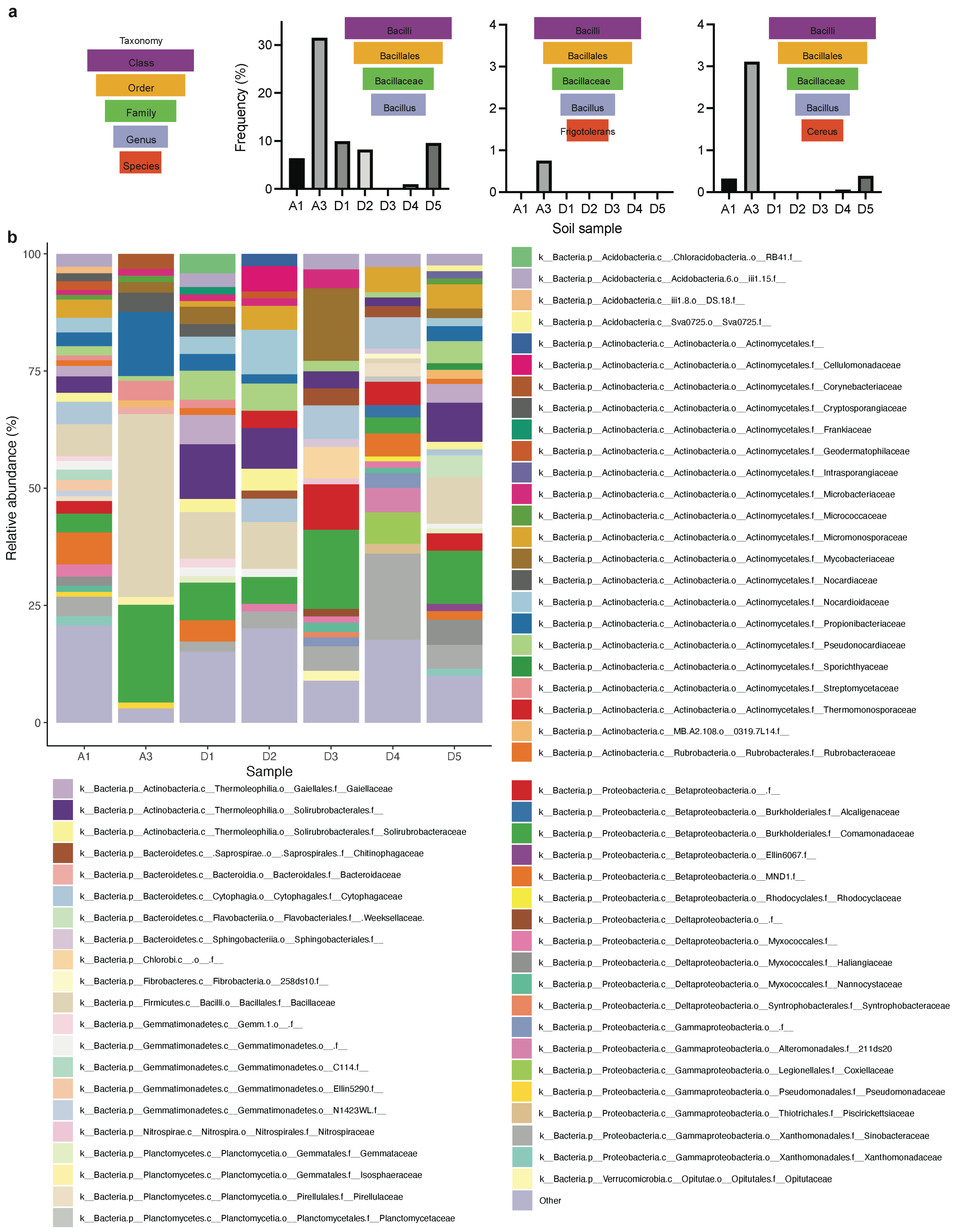

S5 Fig. Quantification of engineered isolates in environmental samples and family-level 16S analysis. (a) Bar graphs represent the relative abundance of the *Bacillus* genus and the *B. frigoritolerans* and *B. cereus* isolates in the soil and surface water samples. Data were obtained from the 16S metagenomic analysis of the samples. The bar pyramid represents the taxonomy genus and isolates presented in each plot. **(b)** 16S rDNA gene sequencing analysis data of soil samples showing the family-level relative abundance. The 'Other' category includes all phyla with relative abundance <1%.

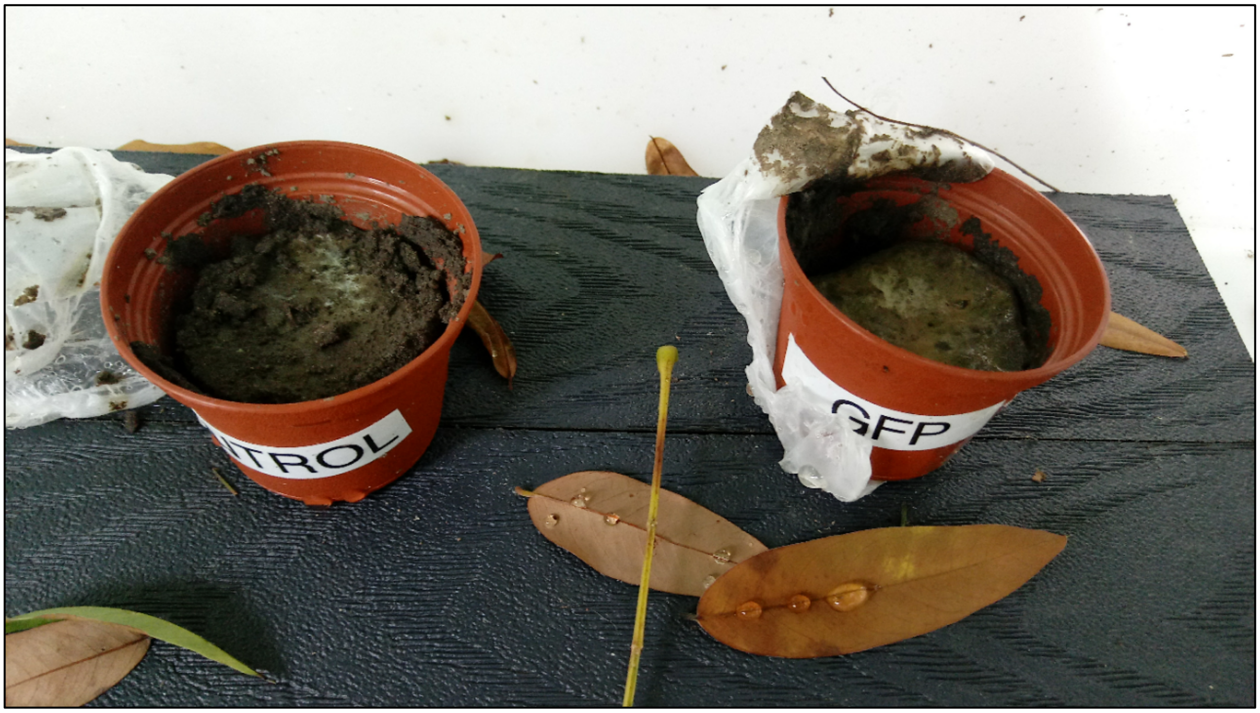

S6 Fig. Image of bacteria-infused soil during simulated environment experiment. Picture of WT (left) and engineered (right) soil infused with *B. frigoritolerans* A3E1 taken on day 6 of the experiment showing the early stages of the mold contamination. The mold was first detected on day 4 of the experiment.

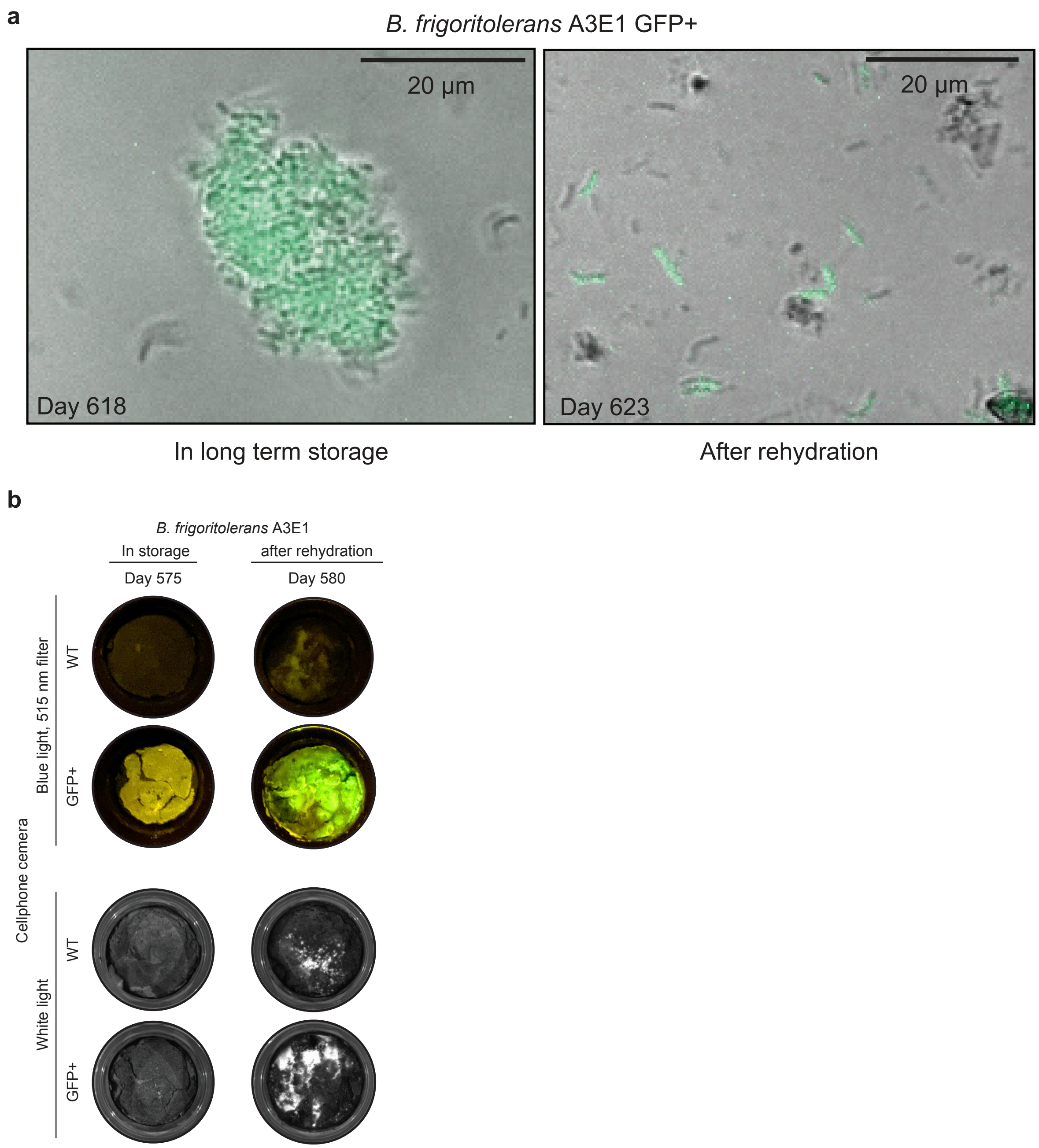

S7 Fig. Imaging of long-storage *B. frigotolerans* A3E1 bacteria-infused soil before and after rehydration. **(a)** Fluorescent microscopy images of GFP+ *B. frigoritolerans* A3E1 samples before (left) and after rehydration (right). The results revealed fluorescent spore-like bacterial clusters, about 1 µm in length, which agrees with known spore sizes[3]. These clusters disappear upon rehydration and transform into rod-shaped bacteria, 4-10 µm in length, similar to *B. subtilis* cells[4]. In addition to the fact that the *B. frigoritolerans* are known to sporulate[1]. **(b)** Wildtype and GFP+ *B. frigoritolerans* A3E1 containing pots in long-term incubation in dry conditions at room temperature were imaged using cell phone cameras under Blue LED flashlight illumination (450 nm) and amber UV protection goggles (top) and white light (bottom).

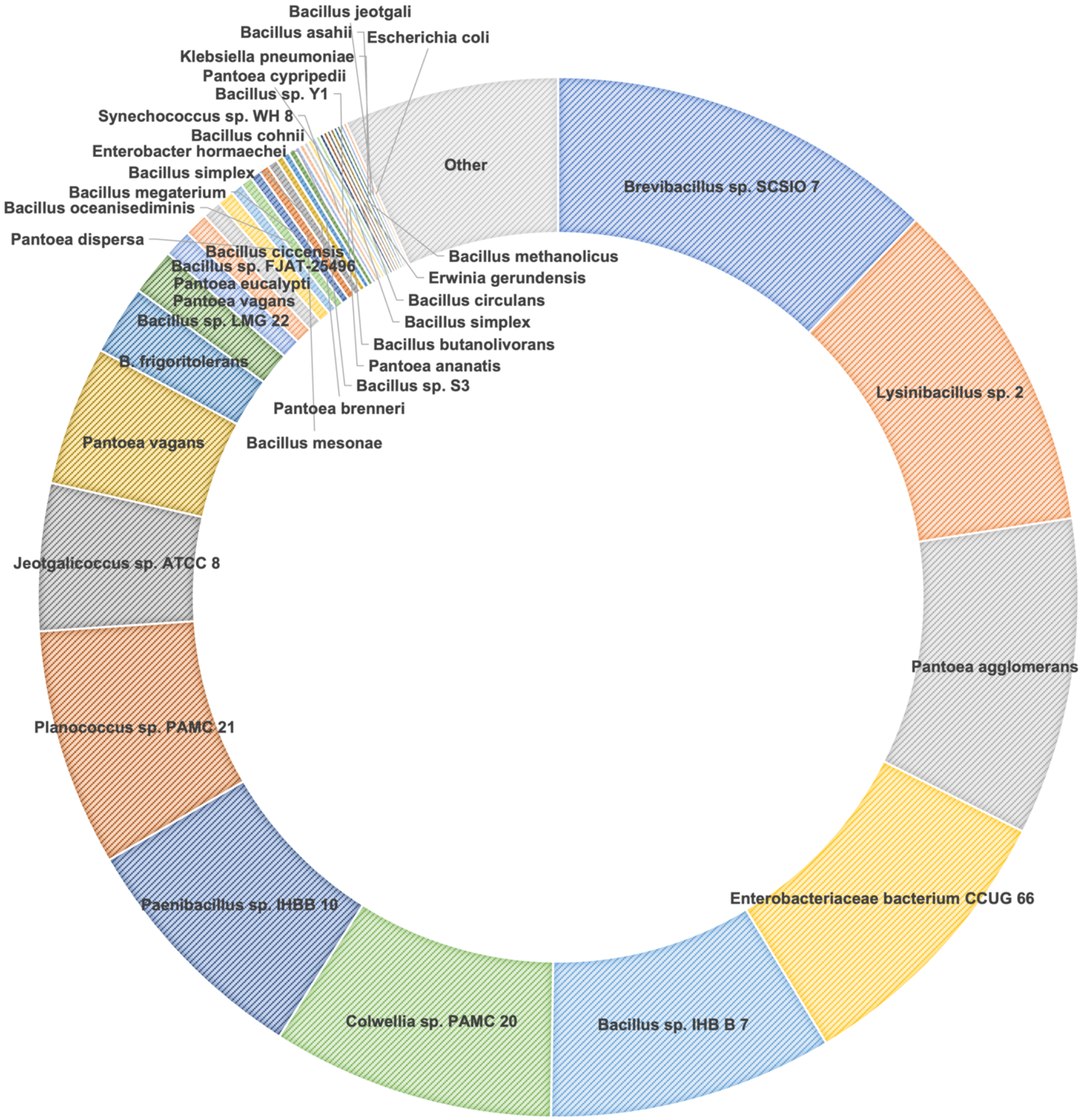

S8 Fig. Metagenomic abundance of bacteria that colonized the soil throughout the long storage. The pie chart represents the relative abundance of bacterial isolates in soil samples. Abundance was calculated from the total length of contigs that belong to each isolate in the *B. frigoritolerans* A3E1 WT long-stored pot. *Bacillus frigoritolerans* was previously annotated as *Brevibacterium* (its former taxonomic name)[1]. Notably, the results were acquired after overnight outgrowth in rich media (LB), and the quantification is not representative of actual abundance in the original soil sample.

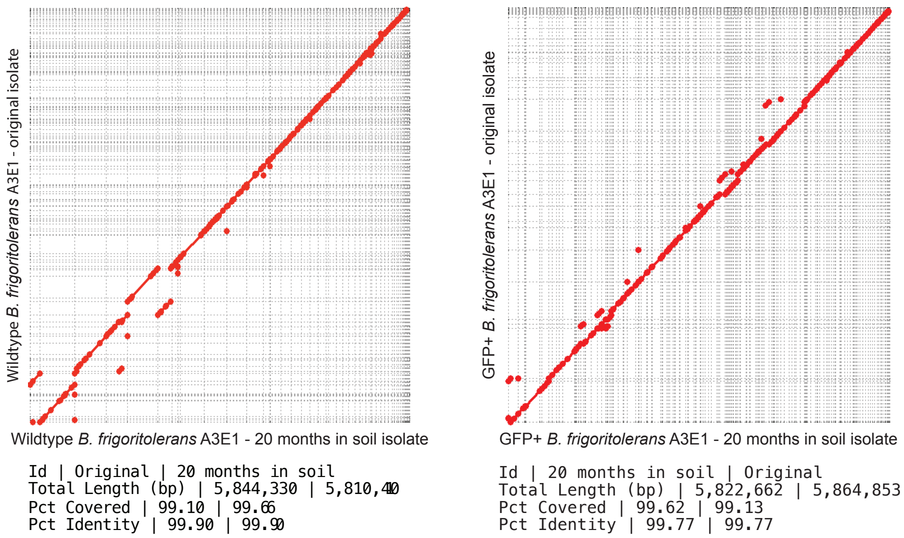

S9 Fig. Comparing genomic assembly before and after long-term soil incubation. Comparison of *B. frigoritolerans* A3E1 WT (left) and GFP+ (right) with progenitor assemblies before soil incubation. The MUMmer plots present the novel undomesticated bacteria shotgun genome assembly on the Y-axis compared with their original progenitor novel assembly on the X-axis. Each horizontal and vertical line represents one assembly contig of the novel assemblies—below each plot is the total length of the assembly, the reciprocal percentages of coverage, and identity.

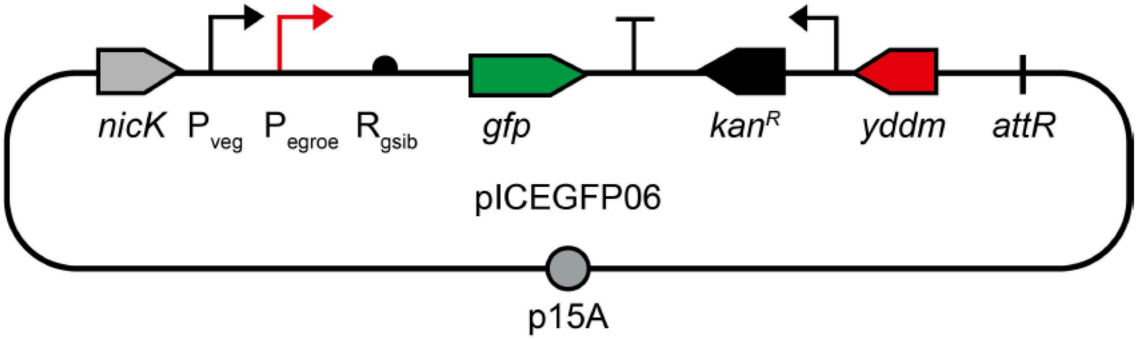

S10 Fig. Payload plasmid map. Adapted from Meng et al.[5]

Supporting methods

16S rRNA gene sequencing analysis of soil samples

The method for DNA extraction from soil was adapted from previously published studies[6,7]. From all soil samples, 3 g of soil were washed three times with 3 mL TE buffer (50 mM Tris-HCL (Sigma 5941), 50mM EDTA (Affymetrix|USB, Cleveland, OH, #15697), pH 8.0. Samples were frozen using liquid nitrogen and macerated by mortar and pestle to a fine powder. Next, 1 g of each sample was moved to 15 mL sterile tubes (VWR #89039), and 2 mL of TE buffer and 2 mL of phenol-chloroform-isoamyl alcohol (25:24:1; Sigma #P3803) were added. The mixture was gently vortexed for 1 min. Next, the mixture was centrifuged at 2700 g for 10 min at 4°C (Eppendorf 5910R). Isopropanol precipitation was performed, followed by DNA clean and concentrator 5 kit (Zymo Research, Irvine, CA, #D4004) to yield metagenomic DNA. To reduce the concentration of PCR inhibitors, samples were 10-fold diluted in water before 16S rDNA amplification PCR. The V3 region of the 16S rDNA was amplified using 337F (CTTTCCCTACACGACGCTCTTCCGATCTGACTCCTACGGGAGGCWGCAG) and 517R (TTCAGACGTGTGCTCTTCCGATCTGTATTACCGCGGCTGCTGG) universal primers that were constructed to contain Illumina adapters overhangs. The Q5 polymerase master mix (NEB #M0515) PCR amplification procedure was the following: anneal at 62°C for 30 s, extend at 72°C for 60 s, for 25 cycles. Library construction for NGS sequencing was done in a second PCR round using the forward primer (AATGATACGGCGACCACCGAGATCTACACTCTTTCCCTACACGACGCTC) and seven reverse primers, containing a different index for each soil sample (GATCGGAAGAGCACACGTCTGAACTCCAGTCAC***NNNNNN***GAATCTCGTATGCCGTCTTCTGCTTG; index region marked in bold). The amplification primers which targeted the V3 region of the 16S rDNA gene across the different bacteria in the soil samples were amplified and were gel-purified using a 1% agarose gel and gel-extraction kit (Zymo Research #D4002) with an amplicon size of around 302 bp. Amplicons were measured using a bioanalyzer DNA high-sensitivity kit (Agilent, Santa Clara, CA) and diluted and pooled to a final concentration of 2 nM for sequencing (Illumina, San Diego, CA, HiSeq 2500). The V3 region was sequenced with 220 bp pair-end reads.

The following was performed for data analysis quality control. Removal of lower sequencing quality bases at the 5' end of the sequences was evaluated using the Illumina QC files. This resulted in the removal of the first 15 nucleotides from each sequence using the fastxtoolkit/0.0.13 short sequence trimming package. Read1 (forward reads) and read2 (reverse reads) from the sequencer were merged using pear[8]/0.9.10 pair-end merger package. Non-biological sequences were removed at this step. 16S

rDNA analysis was performed using qiime2 (q2cli version 2021.2.0)[9]. The fastq files were imported into Qiime2 with the qiime tools import method. Next, using the dada2 plugin[10], quality filtering and chimera removal were performed, and all sequences were trimmed to 150bp. Non-redundant sequences and the count artifacts were created and converted to plain text files using the qiime tools export and biom convert methods. GreenGenes 97 reference sequences and taxonomy data were imported using the qiime tools import method. Closed reference clustering was performed with the qiime vsearch cluster-features-closed-reference method. We have removed the Enterobacteriaceae family (*E. coli, a common lab contaminant*) from the dataset as high and consistent levels of contamination was detected across samples. Bar plot visualization was generated in R using the ggplot2/3.3.3 package[11].

16S rDNA phylogeny of undomesticated soil bacteria

Each conjugated isolate was plated overnight on LB agar plates supplemented with kanamycin at 37°C. The next day, single colonies were picked and lysed according to manufacturer protocol using 35 µL InstaGene Matrix (Bio-rad), 1 µL of the lysis supernatant was used as the PCR template. The 16S rDNA genes were PCR amplified using ReadyMade Primers (IDT, Coralville, IA catalog# 51-01-19-06, 51-01-19-07). The amplicons were sent for Sanger sequencing. The resulting sequences were trimmed and analyzed using EZBioCloud[12]. All sequences are available in Supplementary Table 3. The phylogenetic tree was generated using Phylogeny.fr[13] with default parameters.

Whole-genome sequencing

All undomesticated soil isolates were streaked from glycerol stocks onto LB agar plates supplemented with kanamycin (except for the WT strains of A1E2 and A3E1) and incubated overnight at 37°C. One colony was picked for each isolate and incubated in 3 mL of LB for overnight incubation at 37°C and 250 rpm in 14 mL culture tubes. The resulting cultures were used for genome extraction. Metagenomic sequencing of the long-term storage soil flowerpot was done by taking a small 10 mg soil fragment into 3 mL of LB for overnight incubation at 37°C and 250 rpm in 14 mL culture tubes. The resulting culture was used for genome extraction. The extraction and purification of genomic DNA were performed using the Wizard genomic DNA purification kit (Promega, Madison, WI, A2920). Lysozyme (0.4 mg/mL final concentration) was incubated for 10-20 mins at 37°C. Genome cleavage and tagging (tagmentation) and library preparation were done using the DNA Prep (M) tagmentation kit (Illumina 20018704) and the Nextera™ DNA CD Indexes (Illumina), according to manufacturer protocol. Indexed libraries were run in a HiSeq sequencer using 220bp pair-end reads (Illumina). The identity of each sample's closest reference species

was determined using the Kraken software package[14] using the default Kraken2 database built from RefSeq genomes accessed through the command "kraken2-build --standard". Draft scaffolds were assembled using the SPAdes de-Novo assembler (v. 3.14.1) for isolates and the MetaSPAdes de-novo assembler (v. 3.14) for metagenomic samples (*i.e.*, for the 20-months old soil samples), using default parameters in both cases[15,16]. Each assembly was polished using the Pilon package with "--fix all"[17]. Finally, synteny was computed using the MUMer3.0 package[18]. Synteny plots comparing the assembly for each sample to its closest reference in RefSeq were generated using the command "nucmer -p nucmer," followed by "mummerplot."

Genomic sequencing of the soil samples after the long-term storage experiment were done by taking a small 10 mg soil fragment from the surface soil in each pot and incubating it in 3 mL of LB for 3 hours incubation at 37°C and 250 rpm in 14 mL culture tubes. For the sample taken from the GFP+ pot, kanamycin was supplemented. After incubation, 50 µL of each were spread on LB agar plates and incubated overnight at 37°C. The next day, *B. frigoritolerans* A3E1 colonies dominated the plates, even in the WT case, and one colony was picked and incubated in 3 mL of LB for overnight incubation at 37°C and 250 rpm in 14 mL culture tubes. The genomes of the resulting cultures were extracted using the Wizard genomic DNA purification kit (Promega) under manufacturer protocol. Purified genomes were sent for library preparation at the MIT MicroBio core, which used a DNA prep kit (Illumina 20018705), and Nextera Flex indexes (Illumina 20018708) under manufacturer protocol. Sequencing was done with a final library concentration of 15 pM using MiSeq (Illumina; MiSeq Reagent Kit v3, 600-cycle, MS-102-3003). Pairwise mutations were identified by comparing the raw sequencer reads to the genomic assembly of *B. frigoritolerans* A3E1 done before long-term storage using the breseq[19] tool. We listed mutations that affected an annotated gene (Supplementary Fig. 8). To avoid listing sequencing artifacts, we listed only mutations where the raw reads covering the mutations have at least 10X depth and pure coverage (>90%). In addition, to discount mutations that are a result of sequencing errors in the assembly of the original *B. frigoritolerans* A3E1 genome, all mutations were checked against the closest reference, *B. frigoritolerans* DSM-8801 (GCF_001636405.1), and any mutation that was identical to it was removed from the list.

Fluorescent microscopy of soil samples

Soil samples (1–5 mg) containing bacteria were taken from dry and rehydrated pots. The soil was vortexed in 50 µL PBS for 1 minute. Small soil particles resuspended in 10 µL PBS were immobilized on glass slides for microscopy, using agarose (Lonza, Basel, Switzerland, Seaplaque agarose, 50004) patches prepared as described elsewhere[20]. Fluorescent imaging was performed using an Observer z1 microscope (Zeiss), using

x20 and x40 magnifications with the EGFP fluorescence setting (509 nm) and 30 ms exposure time.

Supporting tables

S1 Table. Undomesticated soil bacteria isolated in this study.

Strain[a]	Closest reference species	NCBI accession #	miniICE found?	Genomic loci[b]	Adjacent to tRNA-Leu ?	Doubling time ± SD (min)	GFP flu' median (au)[c]
**A1E2_WT**	*Brevibacterium frigoritolerans*	SAMN2748119	No			27 ± 9	101 (no GFP)
**A3E1_WT**	*Brevibacterium frigoritolerans*	SAMN2748117	No			22 ± 9	134 (no GFP)
**A1E2**	*Brevibacterium frigoritolerans*	SAMN2748116	Yes	5.4M/5.5M	Yes	31 ± 12	14,722
**A3E1**	*Brevibacterium frigoritolerans*	SAMN2748118	Yes	5.4M/5.5M	Yes	26 ± 10	12,025
**A3E1_WT after LTS[d]**	*Brevibacterium frigoritolerans*	SAMN27734919	Yes	5.4M/5.5M	Yes		
**A3I1**	*Bacillus paralicheniformis*	SAMN2748123	Yes	0.1M/4.4M	No	40 ± 17	3,191
**D1A1**	*Bacillus pumilus*	SAMN2748121	Yes	0.5M/3.7M	Yes	28 ± 9	1,506
**D1B1**	*Bacillus velezensis*	SAMN2748124	Yes	0.5M/4M	Yes	33 ± 7	2,801
**D1B2**	*Lysinibacillus macroides*	SAMN2748135	Yes	4.1M/4.6M	Yes	43 ± 8	2,257
**D2A1**	*Lysinibacillus sphaericus*	SAMN2748126	Yes	0.2M/4.5M	No	32 ± 8	2,464
**D2A2**	*Lysinibacillus sphaericus*	SAMN2748131	Yes	0.5M/4.6M	Yes	32 ± 6	4,160
**D3A2**	*Bacillus cereus*	SAMN2748120	Yes	0.3M/5.3M	No	34 ± 14	371
**D3B1**	*Lysinibacillus sphaericus*	SAMN2748129	Yes	0.2M/4.5M	No	35 ± 8	3,735
**D3B2**	*Lysinibacillus sphaericus*	SAMN2748134	?	--/4.5M	No	38 ± 11	4,542
**D3C1**	*Bacillus cereus*	SAMN2748130	Yes	0.2M/5.4M	Yes	26 ± 9	416
**D3C2**	*Lysinibacillus*	SAMN2748127	Yes	4.1M/4.6M	Yes	44 ± 12	4,371
**D3C4**	*Bacillus paralicheniformis*	SAMN27734920	Yes	2.9M/4.4M	No	28 ± 12	2,711
**D4A1**	*Lysinibacillus*	SAMN2748122	Yes	4.1M/4.6M	Yes	30 ± 8	1,860
**D4A2**	*Lysinibacillus*	SAMN2748132	Yes	4.1M/4.6M	Yes	28 ± 5	1,907
**D4A3**	*Lysinibacillus*	SAMN2748125	Yes	4.1M/ 4.6M	Yes	28 ± 5	3,803
**D4B1**	*Lysinibacillus*	SAMN2748136	Yes	4.1M/4.6M	Yes	28 ± 6	2,720
**D4B2**	*Lysinibacillus*	SAMN2748128	Yes	4.1M/4.6M	Yes	29 ± 8	1,941
**D4B3**	*Lysinibacillus*	SAMN2748137	Yes	4.1M/4.6M	Yes	35 ± 12	4,050
**D4C1**	*Bacillus cereus*	SAMN27734921	Yes	0.2M/ 5.5M	Yes	28 ± 10	9,684
**D4C2**	*Bacillus cereus*	SAMN27734922	Yes	0.2M/ 5.5M	Yes	19 ± 3	4,258
**D4C3**	*Lysinibacillus*	SAMN27734923	Yes	4.5M/ 5.3M	Yes	21 ± 2	8,197
**D4C4**	*Bacillus cereus*	SAMN27734924	Yes	0.2M/ 5.5M	Yes	22 ± 3	2,873
**D5B1**	*Bacillus cereus*	SAMN2748133	Yes	0.2M/5.4M	Yes	30 ± 13	467

a. Each isolate is referred to as a concatenation of the isolation location and the sample name
b. Location in the closest annotated reference genome / closest annotated reference genome size
b. Measured using flow cytometry
c. LTS – Long-term storage

S2 Table. Genetic part sequences

Name	Class	DNA sequence
gfp	gene	ATGAAAGGAGAAGAACTTTTCACTGGAGTTGTCCCAATTCTTGTTGAATTAGATGGTGATGTTA<br>ATGGGCACAAATTTTCTGTCAGTGGAGAGGGTGAAGGTGATGCAACATACGGAAAACTTACCC<br>TTAAATTTATTTGCACTACTGGAAAACTACCTGTTCCATGGCCAACACTTGTCACTACTTTCGCGT<br>ATGGTCTTCAATGCTTTGCGAGATACCCAGATCATATGAAACAGCATGACTTTTTCAAGAGTGC<br>CATGCCCGAAGGTTATGTACAGGAAAGAACTATATTTTTCAAAGATGACGGGAACTACAAGAC<br>ACGTGCTGAAGTCAAGTTTGAAGGTGATACCCTTGTTAATAGAATCGAGTTAAAAGGTATTGAT<br>TTTAAAGAAGATGGAAACATTCTTGGACACAAATTGGAATACAACTATAACTCACACAATGTAT<br>ACATCATGGCAGACAAACAAAAGAATGGAATCAAAGTTAACTTCAAAATTAGACACAACATTG<br>AAGATGGAAGCGTTCAACTAGCAGACCATTATCAACAAAATACTCCAATTGGCGATGGCCCTGT<br>CCTTTTACCAGACAACCATTACCTGTCCACACAATCTAAGCTTTCGAAAGATCCCAACGAAAAGA<br>GAGACCACATGGTCCTTCTTGAGTTTGTAACAGCTGCTGGGATTACACATGGCATGGATGAACT<br>ATACAAATAA
$^{P}{}_{veg}$	promoter	AATTTTGTCAAAATAATTTTATTGACAACGTCTTATTAACGTTGATATAATTTAAATTTTATTTGA<br>CAAAAATGGGCTCGTGTTGTACAATAAATGT
P_{egroe}	promoter	CAGCTATTGTAACATAATCGGTACGGGGGTGAAAAAGCTAACGGAAAAGGGAGCGGAAAAGA<br>ATGATGTAAGCGTGAAAAATTTTTTAAAAAA<br>TCTCTTGACATTGGAAGGGAGATATGTTATTATAAGAATT
R_{gsib}	RBS	TAAAGGAGGAATTCAAA
	Double terminator	TACCTAGATTTAGATGTCTAAAAAGCTTTAACTACAAGCTTTTTAGACATCTAATCTTTTCTGTTT<br>TTTCGGAAGGAAATGATGACCTCGTTTCCACCGAATTAGCTTGCATGCGGCTAGCTTA

S3 Table: 16S sequences of isolated undomesticated soil bacteria.

> *Bacillus frigoritolerans* A1E2
NCCNNNNCTGTCCACCTTAGGCGGCTGGCTCCATGAAGGTTACCTCACCGACTTCGGGTGTTACAAACTCTCGTGGTGTGACGGGCGGTGTGTACAAGGCCCG
GGAACGTATTCACCGCGGCATGCTGATCCGCGATTACTAGCGATTCCGGCTTCATGCAGGCGAGTTGCAGCCTGCAATCCGAACTGAGAATGGCTTTATGGGAT
TCGCTTACCTTCGCAGGTTTGCAGCCCTTTGTACCATCCATTGTAGCACGTGTGTAGCCCAGGTCATAAGGGGCATGATGATTTGACGTCATCCCCACCTTCCTCC
GGTTTGTCACCGGCAGTCACCTTAGAGTGCCCAACTGAATGCTGGCAACTAAGATCAAGGGTTGCGCTCGTTGCGGGACTTAACCCAACATCTCACGACACGAG
CTGACGACAACCATGCACCACCTGTCACTCTGTCCCCCGAAGGGGAAAGCCCTATCTCTAGGGTTGTCAGAGGATGTCAAGACCTGGTAAGGTTCTTCGCGTTG
CTTCGAATTAAACCACATGCTCCACCGCTTGTGCGGGCCCCCGTCAATTCCTTTGAGTTTCAGCCTTGCGGCCGTACTCCCCAGGCGGAGTGCTTAATGCGTTAGC
TGCAGCACTAAAGGGCGGAAACCCTCTAACACTTAGCACTCATCGTTTACGGCGTGGACTACCAGGGTATCTAATCCTGTTTGCTCCCCACGCTTTCGCGCCTCA
GTGTCAGTTACAGACCAGAAAGTCGCCTTCGCCACTGGTGTTCCTCCAAATCTCTACGCATTTCACCGCTACACTTGGAATTCCACTTTCCTCTTCTGCACTCAAGT
TCCCCAGTTTCCAATGACCCTCCACGGTTGAGCCGTGGGCTTTCACATCAGACTTAAAGGAACCACCTGCGCGCGCTTTACGCCCAATAATTCCGGACAACGCTT
GCCACCTACGTATTACCGCGGCTGCTGGCACGTAGTTAGCCGTGGCTTTCTGGTTAGGGTACCGTCAAGGTACCCAGCAGTTACTCTGGTACCTGG

> *Bacillus frigoritolerans* A3E1
NCCANNTCTGTCCACCTTAGGCGGCTGGCTCCATGAAGGTTACCTCACCGACTTCGGGTGTTACAAACTCTCGTGGTGTGACGGGCGGTGTGTACAAGGCCCGG
GAACGTATTCACCGCGGCATGCTGATCCGCGATTACTAGCGATTCCGGCTTCATGCAGGCGAGTTGCAGCCTGCAATCCGAACTGAGAATGGCTTTATGGGATT
CGCTTACCTTCGCAGGTTTGCAGCCCTTTGTACCATCCATTGTAGCACGTGTGTAGCCCAGGTCATAAGGGGCATGATGATTTGACGTCATCCCCACCTTCCTCCG
GTTTGTCACCGGCAGTCACCTTAGAGTGCCCAACTGAATGCTGGCAACTAAGATCAAGGGTTGCGCTCGTTGCGGGACTTAACCCAACATCTCACGACACGAGC
TGACGACAACCATGCACCACCTGTCACTCTGTCCCCCGAAGGGGAAAGCCCTATCTCTAGGGTTGTCAGAGGATGTCAAGACCTGGTAAGGTTCTTCGCGTTGC
TTCGAATTAAACCACATGCTCCACCGCTTGTGCGGGCCCCCGTCAATTCCTTTGAGTTTCAGCCTTGCGGCCGTACTCCCCAGGCGGAGTGCTTAATGCGTTAGCT
GCAGCACTAAAGGGCGGAAACCCTCTAACACTTAGCACTCATCGTTTACGGCGTGGACTACCAGGGTATCTAATCCTGTTTGCTCCCCACGCTTTCGCGCCTCAG
TGTCAGTTACAGACCAGAAAGTCGCCTTCGCCACTGGTGTTCCTCCAAATCTCTACGCATTTCACCGCTACACTTGGAATTCCACTTTCCTCTTCTGCACTCAAGTT
CCCCAGTTTCCAATGACCCTCCACGGTTGAGCCGTGGGCTTTCACATCAGACTTAAGGAACCACCTGCGCGCGCTTTACGCCCAATAATTCCGGGACAACGCTTG
CCACCTACGTATTACCGCGGCTGCTGGCACGTAGTTAGCCGTGGCTTTCTGGTTAGGTACCGTCAAGGTACCAGCAGTTACTCTGGNACTTTNNTTCTTNN

> *Bacillus paralicheniformis* A3I1
CNNANCTGTCCACCTTCGGCGGCTGGCTCCAAAAGGTTACCTCACCGACTTCGGGTGTTACAAACTCTCGTGGTGTGACGGGCGGTGTGTACAAGGCCCGGGA
ACGTATTCACCGCGGCATGCTGATCCGCGATTACTAGCGATTCCAGCTTCACGCAGTCGAGTTGCAGACTGCGATCCGAACTGAGAACAGATTTGTGGGATTGG
CTTAGCCTCGCGGCTTCGCTGCCCTTTGTTCTGCCCATTGTAGCACGTGTGTAGCCCAGGTCATAAGGGGCATGATGATTTGACGTCATCCCCACCTTCCTCCGGT
TTGTCACCGGCAGTCACCTTAGAGTGCCCAACTGAATGCTGGCAACTAAGATCAAGGGTTGCGCTCGTTGCGGGACTTAACCCAACATCTCACGACACGAGCTG
ACGACAACCATGCACCACCTGTCACTCTGCCCCCGAAGGGGAAGCCCTATCTCTAGGGTTGTCAGAGGATGTCAAGACCTGGTAAGGTTCTTCGCGTTGCTTCG
AATTAAACCACATGCTCCACCGCTTGTGCGGGCCCCCGTCAATTCCTTTGAGTTTCAGTCTTGCGACCGTACTCCCCAGGCGGAGTGCTTAATGCGTTTGCTGCAG
CACTAAAGGGCGGAAACCCTCTAACACTTAGCACTCATCGTTTACGGCGTGGACTACCAGGGTATCTAATCCTGTTCGCTCCCCACGCTTTCGCGCCTCAGCGTC
AGTTACAGACCAGAGAGTCGCCTTCGCCACTGGTGTTCCTCCACATCTCTACGCATTTCACCGCTACACGTGGAATTCCACTCTCCTCTTCTGCACTCAAGTTCCCC
AGTTTCCAATGACCCCTCCCCCGGGTTGAGGCCGGGGGGCTTTCAACATCAAAACTTAAAAAAACCCGCCCTGGCGCGCCCGCCTTTACGCCCCAATAAATTCCG
GGANCAAACGCTTTGCCACCCTAACGTATTTAACCCGCCGGCCTGCCNGGNCACGTANNNANCCGTGGNNTTNTGNNNNGGTANNGTCNNNACNGCCNATC
NACGGTACCTGGTNNNCTACANNNNNNANGATCCNNAACCTNNCTCACGCGCNNNCCGTNNANTNNNNCATGNNNNNNNNNGTGC

> *Bacillus pumilus* D1A1
CCNNCTGTCCACCTTCGGCGGCTGGCTCCATAAAGGTTACCTCACCGACTTCGGGTGTTACAAACTCTCGTGGTGTGACGGGCGGTGTGTACAAGGCCCGGGAA
CGTATTCACCGCGGCATGCTGATCCGCGATTACTAGCGATTCCAGCTTCACGCAGTCGAGTTGCAGACTGCGATCCGAACTGAGAACAGATTTATGGGATTGGC
TAAACCTTGCGGTCTCGCAGCCCTTTGTTCTGTCCATTGTAGCACGTGTGTAGCCCAGGTCATAAGGGGCATGATGATTTGACGTCATCCCCACCTTCCTCCGGTT
TGTCACCGGCAGTCACCTTAGAGTGCCCAACTAAATGCTGGCAACTAAGATCAAGGGTTGCGCTCGTTGCGGGACTTAACCCAACATCTCACGACACGAGCTGA
CGACAACCATGCACCACCTGTCACTCTGTCCCCGAAGGGAAAGCCCTATCTCTAGGGTTGTCAGAGGATGTCAAGACCTGGTAAGGTTCTTCGCGTTGCTTCGAA
TTAAACCACATGCTCCACCGCTTGTGCGGGCCCCCGTCAATTCCTTTGAGTTTCAGTCTTGCGACCGTACTCCCCAGGCGGAGTGCTTAATGCGTTAGCTGCAGC
ACTAAGGGGCGGAAACCCCCTAACACTTAGCACTCATCGTTTACGGCGTGGACTACCAGGGTATCTAATCCTGTTCGCTCCCCACGCTTTCGCTCCTCAGCGTCA
GTTACAGACCAGAGAGTCGCCTTCGCCACTGGTGTTCCTCCACATCTCTACGCATTTCACCGCTACACGTGGAATTCCACTCTCCTCTTCTGCACTCAAGTTTCCCA
GTTTCCAATGACCCTCCCCGGTTGAGCCGGGGGCTTTCACATCAGACTTAAGAAACCGCCTGCGAGCCCTTTACGCTCAATAATTCCGGACAACGCTTGCCACC
TACGTATTACCGCGGCTGCTGGCACGTAGTTAGCCGTGGCTTTCTGGNTANGTGCCGTCAAGTGCGAGCAGTACTCTCGCACTTGGTTCTGCTCGTACACAGNA
GCTNTACGAGNCCNNNNNAACGTCAANNACTCACGNNGCNGTNCCTNGGTCTGACNTTCGTTCCANTGGCGGNATCANNNGTANTGACTNNNNNNNGTTA
GCAGTCTNGGAANNCNGATGAGTGACTGNNNGNNNN

> *Bacillus velezansis* D1B1
CNNCNCTGTCCACCTTCGGCGGCTGGCTCCATAAAAGGTTACCTCACCGACTTCGGGTGTTACAAACTCTCGTGGTGTGACGGGCGGTGTGTACAAGGCCCGG
GAACGTATTCACCGCGGCATGCTGATCCGCGATTACTAGCGATTCCAGCTTCACGCAGTCGAGTTGCAGACTGCGATCCGAACTGAGAACAGATTTGTGGGATT
GGCTTAACCTCGCGGTTTCGCTGCCCTTTGTTCTGTCCATTGTAGCACGTGTGTAGCCCAGGTCATAAGGGGCATGATGATTTGACGTCATCCCCACCTTCCTCCG
GTTTGTCACCGGCAGTCACCTTAGAGTGCCCAACTGAATGCTGGCAACTAAGATCAAGGGTTGCGCTCGTTGCGGGACTTAACCCAACATCTCACGACACGAGC
TGACGACAACCATGCACCACCTGTCACTCTGCCCCCGAAGGGGACGTCCTATCTCTAGGATTGTCAGAGGATGTCAAGACCTGGTAAGGTTCTTCGCGTTGCTTC
GAATTAAACCACATGCTCCACCGCTTGTGCGGGCCCCCGTCAATTCCTTTGAGTTTCAGTCTTGCGACCGTACTCCCCAGGCGGAGTGCTTAATGCGTTAGCTGC
AGCACTAAGGGGCGGAAACCCCCTAACACTTAGCACTCATCGTTTACGGCGTGGACTACCAGGGTATCTAATCCTGTTCGCTCCCCACGCTTTCGCTCCTCAGCG
TCAGTTACAGACCAGAGAGTCGCCTTCGCCACTGGTGTTCCTCCACATCTCTACGCATTTCACCGCTACACGTGGAATTCCACTCTCCTCTTCTGCACTCAAGTTCC
CCAGTTTCCAATGACCCTTCCCCGGTTGAGCCGGGGGCTTTCACATCAAACCTTAAGAAAACAGCCCGGCGAGCCCATTTACTGCCCAGNCAAATTCCGGTN
NAACGACTTGGCCATCCTACCGTATTANTATCNGCCTGGCTGGCCACNNANNAGCCCGTAGCCTTTCCTGGTTTAAGNGACNGTCACGGTGCAGGCCCTANNN

NCNAACNGGNANNNGNTCNNTGCCNTACCTCACNNNANNNTNNNGGATNCTNANNNNNNNNNNNNNCNACNGNNNNGCTCGNTNGACTTNNNGCNNNN
TNNNNNNNNNTNNNGTACGTAGCNTCGNNNNNNNNNN

> ***Lysinibacillus macroides*** **D1B2**
CCNANCTATCCACCTNTCGGCGGCTGGCTCCAAAAGGTTACCTCACCGACTTCGGGTGTTACAAACTCTCGTGGTGTGACGGGCGGTGTGTACAAGGCCCGGG
AACGTATTCACCGCGGCATGCTGATCCGCGATTACTAGCGATTCCGGCTTCATGTAGGCGAGTTGCAGCCTACAATCCGAACTGAGAACGACTTTATCGGATTA
GCTCCCTCTCGCGAGTTGGCAACCGTTTGTATCGTCCATTGTAGCACGTGTGTAGCCCAGGTCATAAGGGGCATGATGATTTGACGTCATCCCCACCTTCCTCCG
GTTTGTCACCGGCAGTCACCTTAGAGTGCCCAACTAAATGATGGCAACTAAGATCAAGGGTTGCGCTCGTTGCGGGACTTAACCCAACATCTCACGACACGAGC
TGACGACAACCATGCACCACCTGTCACCGTTGCCCCCGAAGGGGAAACTATATCTCTACAGTGGTCAACGGGATGTCAAGACCTGGTAAGGTTCTTCGCGTTGC
TTCGAATTAAACCACATGCTCCACCGCTTGTGCGGGCCCCCGTCAATTCCTTTGAGTTTCAGTCTTGCGACCGTACTCCCCAGGCGGAGTGCTTAATGCGTTAGCT
GCAGCACTAAGGGGCGGAAACCCCCTAACACTTAGCACTCATCGTTTACGGCGTGGACTACCAGGGTATCTAATCCTGTTTGCTCCCCACGCTTTCGCGCCTCAG
CGTCAGTTACAGACCAGAAAGTCGCCTTCGCCACTGGTGTTCCTCCAAATCTCTACGCATTTCACCGCTACACTTGGAATTCCACTTTCCTCTTCTGCACTCAAGTC
CCCCAGTTTCCAATGACCCTCCACGGTTGAGCCGTGGGCTTTCACATCAGACTTAAAGGACCGCCTGCGCGCGCTTTACGCCCAATAATTCCGGACAACGCTTGC
CACCTACGTATTACCGCGGCTGCTGGCACGTAGTTAGCCGTGGCTTTCTAATAANGTACCNGTCAGGTACAGGCCAGTTACTACTGTACTTTGTTCTTCCCTTACA
NAGANTTTTACGATCGAANANNNNTCANTCACGCGCCGTGGCTTCATCAGCTTCGCCCATGNNNNNNNNNNNTACTGCTGCNTCCGTAAGGNNNTCCTN

> ***Lysinibacillus fusiformis*** **D2A1**
CNNANCTATCCACCTTCGGCGGCTGGCTCCAAAAGGTTACCTCACCGACTTCGGGTGTTACAAACTCTCGTGGTGTGACGGGCGGTGTGTACAAGGCCCGGGA
ACGTATTCACCGCGGCATGCTGATCCGCGATTACTAGCGATTCCGGCTTCATGTAGGCGAGTTGCAGCCTACAATCCGAACTGAGAACGACTTTATCGGATTAGC
TCCCTCTCGCGAGTTGGCAACCGTTTGTATCGTCCATTGTAGCACGTGTGTAGCCCAGGTCATAAGGGGCATGATGATTTGACGTCATCCCCACCTTCCTCCGGTT
TGTCACCGGCAGTCACCTTAGAGTGCCCAACTAAATGATGGCAACTAAGATCAAGGGTTGCGCTCGTTGCGGGACTTAACCCAACATCTCACGACACGAGCTGA
CGACAACCATGCACCACCTGTCACCGTTGCCCCCGAAGGGGAAACCATATCTCTACAGTGGTCAACGGGATGTCAAGACCTGGTAAGGTTCTTCGCGTTGCTTC
GAATTAAACCACATGCTCCACCGCTTGTGCGGGCCCCCGTCAATTCCTTTGAGTTTCAGTCTTGCGACCGTACTCCCCAGGCGGAGTGCTTAATGCGTTAGCTGC
AGCACTAAGGGGCGGAAACCCCCTAACACTTAGCACTCATCGTTTACGGCGTGGACTACCAGGGTATCTAATCCTGTTTGCTCCCCACGCTTTCGCGCCTCAGTG
TCAGTTACAGACCAGATAGTCGCCTTCGCCACTGGTGTTCCTCCAAATCTCTACGCATTTCACCGCTACACTTGGAATTCCACTATCCTCTTCTGCACTCAAGTCTC
CCAGTTTCCAATGACCCTCCACGGTTGAGCCGTGGGCTTTCACATCAGACTTAAGAAACCACCTGCGCGCGCTTTACGCCCAATAATTCCGGACAACGCTTGCCA
CCTACGTATTACCGCGGCTGCTGGCACGTAGTTAGCCGTGGCTTTCTAATACNGTACCGTCANGTACAGCCAGTTACTACTGTACTTGTTCTTCCCTTACANAGA
NTTTTACGAANCCGAATNNTCTNACTCACGCGGCGTTGCTTCATCAAGCTTCGCCCATTGGTNANNNNNNNACTGACTGCCCTNCGTNNNNNNNCNNNNNN
NNNNNCTCGAGTTCNCCANNNNNNN

> ***Lysinibacillus fusiformis*** **D2A2**
CCNANCTATCCACCTTTCGGCGGCTGGCTCCAAAAGGTTACCTCACCGACTTCGGGTGTTACAAACTCTCGTGGTGTGACGGGCGGTGTGTACAAGGCCCGGGA
ACGTATTCACCGCGGCATGCTGATCCGCGATTACTAGCGATTCCGGCTTCATGTAGGCGAGTTGCAGCCTACAATCCGAACTGAGAACGACTTTATCGGATTAGC
TCCCTCTCGCGAGTTGGCAACCGTTTGTATCGTCCATTGTAGCACGTGTGTAGCCCAGGTCATAAGGGGCATGATGATTTGACGTCATCCCCACCTTCCTCCGGTT
TATCACCGGCAGTCACCTTAGAGTGCCCAACTAAATGATGGCAACTAAGATCAAGGGTTGCGCTCGTTGCGGGACTTAACCCAACATCTCACGACACGAGCTGA
CGACAACCATGCACCACCTGTCACCGTTGCCCCCGAAGGGGAAACCATATCTCTACAGTGGTCAACGGGATGTCAAGACCTGGTAAGGTTCTTCGCGTTGCTTC
GAATTAAACCACATGCTCCACCGCTTGTGCGGGCCCCCGTCAATTCCTTTGAGTTTCAGTCTTGCGACCGTACTCCCCAGGCGGAGTGCTTAATGCGTTAGCTGC
AGCACTAAGGGGCGGAAACCCCCTAACACTTAGCACTCATCGTTTACGGCGTGGACTACCAGGGTATCTAATCCTGTTTGCTCCCCACGCTTTCGCGCCTCAGTG
TCAGTTACAGACCAGATAGTCGCCTTCGCCACTGGTGTTCCTCCAAATCTCTACGCATTTCACCGCTACACTTGGTATTCCACTATCCTCTTCTGCACTCAAGTCTC
CCAGTTTCCAATGACCCTCCACGGTTGAGCCGTGCGCTTTCACATCAGACTTAAGAAACCACCTGCGCGCGCTTTACGCCCAATAATTCCGGACAACGCTTGCCA
GCTACGTATTACCGCTGACTGCTGTCACGTAGTTTAGCCGTGAGCTTTGTAATAATGTAGCAGTGCCACGTACAGGGCAGCTACGTACTGTACNTTNTNNTCGN
TTACTACNCNAGCCTCTACGTANTCCGAANNNNNNNCATAACCTCCCNTCGACGGTAGGCGNNNAGTCATGGCCNNTCCGANCGNTTTNNTGGNANNATTG
NNNNAACTGACTTNNNNNCGGGGTGACGCAGNNNNGGGAANNAGNNAGTCCN

> ***Bacillus thuringiensis*** **D3A2**
CCTNNCTGTCCACCTTAGGCGGCTGGCTCCAAAAAGGTTACCCCACCGACTTCGGGTGTTACAAACTCTCGTGGTGTGACGGGCGGTGTGTACAAGGCCCGGG
AACGTATTCACCGCGGCATGCTGATCCGCGATTACTAGCGATTCCAGCTTCATGTAGGCGAGTTGCAGCCTACAATCCGAACTGAGAACGGTTTTATGAGATTA
GCTCCACCTCGCGGTCTTGCAGCTCTTTGTACCGTCCATTGTAGCACGTGTGTAGCCCAGGTCATAAGGGGCATGATGATTTGACGTCATCCCCACCTTCCTCCGG
TTTGTCACCGGCAGTCACCTTAGAGTGCCCAACTTAATGATGGCAACTAAGATCAAGGGTTGCGCTCGTTGCGGGACTTAACCCAACATCTCACGACACGAGCT
GACGACAACCATGCACCACCTGTCACTCTGCTCCCGAAGGAGAAGCCCTATCTCTAGGGTTTTCAGAGGATGTCAAGACCTGGTAAGGTTCTTCGCGTTGCTTCG
AATTAAACCACATGCTCCACCGCTTGTGCGGGCCCCCGTCAATTCCTTTGAGTTTCAGCCTTGCGGCCGTACTCCCCAGGCGGAGTGCTTAATGCGTTAACTTCAT
CACTAAAGGGCGGGAACCGTCTAACACTTAGCACTCATCGTTTACGGCGTGGACTACCAAGGTATCTAATCCTGTTTGCTCCCCACGCTTTCTTGCCTCAGTGTCA
GTTACGGACCAGAGAGTCGAGTTCCCCGCTGCAGTTCCTCTGGATCTCTACGCATTTCAATGGATACGCGTGAAATGTGAAATGGGCTTTTCTGCTGCTTTCGCG
TCTCATTTTTCCCATTTACGCATTCCCGCCTGAGGGAGTGAGGCTGGCACGCTCTCACATCAAAGGAAAATGAACGCGCGGCCCCGCGCAATTCGGTGCACCATT
GTTGCTTGAATATCGATGCGACAGCGAAGGATACTTTACGAGGCCCGTTGACATCGTATGTAGACCGCGTACTATATAGGGNNATGCTACCTTCCGGGAGGCA
GCAGTGANNAGGTCNNCTGCANNGNTTGTCGTCCAGCCTNCGTGACCAGTGANNTATGATNGNNCTNAAATGCCCNTCAATCGAGTCNCCANNGCCTTNATC
TNNNNNAGNANTNNNTNACATTTNNNANANNATACGGTANNNGGGCGGCTNNNCANNANCNNNN

> ***Lysinibacillus fusiformis*** **D3B1**
NCCNANCTATCCACCTTCGGCGGCTGGCTCCAAAAGGTTACCTCACCGACTTCGGGTGTTACAAACTCTCGTGGTGTGACGGGCGGTGTGTACAAGGCCCGGG
AACGTATTCACCGCGGCATGCTGATCCGCGATTACTAGCGATTCCGGCTTCATGTAGGCGAGTTGCAGCCTACAATCCGAACTGAGAACGACTTTATCGGATTA
GCTCCCTCTCGCGAGTTGGCAACCGTTTGTATCGTCCATTGTAGCACGTGTGTAGCCCAGGTCATAAGGGGCATGATGATTTGACGTCATCCCCACCTTCCTCCG
GTTTATCACCGGCAGTCACCTTAGAGTGCCCAACTAAATGATGGCAACTAAGATCAAGGGTTGCGCTCGTTGCGGGACTTAACCCAACATCTCACGACACGAGC
TGACGACAACCATGCACCACCTGTCACCGTTGCCCCCGAAGGGGAAACCATATCTCTACAGTGGTCAACGGGATGTCAAGACCTGGTAAGGTTCTTCGCGTTGC
TTCGAATTAAACCACATGCTCCACCGCTTGTGCGGGCCCCCGTCAATTCCTTTGAGTTTCAGTCTTGCGACCGTACTCCCCAGGCGGAGTGCTTAATGCGTTAGCT
GCAGCACTAAGGGGCGGAAACCCCCTAACACTTAGCACTCATCGTTTACGGCGTGGACTACCAGGGTATCTAATCCTGTTTGCTCCCCACGCTTTCGCGCCTCAG

TGTCAGTTACAGACCAGATAGTCGCCTTCGCCACTGGTGTTCCTCCAAATCTCTACGCATTTCACCGCTACACTTGGAATTCCACTATCCTCTTCTGCACTCAAGTC
TCCCAGTTTCCAATGACCCTCCACGGTTGAGCCGTGGGCTTTCACATCAGACTTAAGAAACCACCTGCGCGCGCTTTACGCCCAATAATTCCGGACAACGCTTGC
CACCTACGTATTACCGCGGCTGCTGGCACGTAGTTAGCCGTGGCTTTCTAATANNNTACCGTCAAGGTACAGCCAGTTACTACTGTACTTTGTTCTTCCCTTACAN
AGANTTTTACGAANCCGAATNNNNNNACTCACGCGGCGTTGCTTCATCANGCTTTTCNCCATNGGTGGANNNNNNACTGCCTGGCTTCCCGTAGGAGTCNGG
GGNNNNNGTGNGNN

> *Lysinibacillus fusiformis* D3B2
CCTNNCTATCCACCTTCGGCGGCTGGCTCCAAAAGGTTACCTCACCGACTTCGGGTGTTACAAACTCTCGTGGTGTGACGGGCGGTGTGTACAAGGCCCGGGAA
CGTATTCACCGCGGCATGCTGATCCGCGATTACTAGCGATTCCGGCTTCATGTAGGCGAGTTGCAGCCTACAATCCGAACTGAGAACGACTTTATCGGATTAGCT
CCCTCTCGCGAGTTGGCAACCGTTTGTATCGTCCATTGTAGCACGTGTGTAGCCCAGGTCATAAGGGGCATGATGATTTGACGTCATCCCCACCTTCCTCCGGTTT
ATCACCGGCAGTCACCTTAGAGTGCCCAACTAAATGATGGCAACTAAGATCAAGGGTTGCGCTCGTTGCGGGACTTAACCCAACATCTCACGACACGAGCTGAC
GACAACCATGCACCACCTGTCACCGTTGCCCCCGAAGGGGAAACCATATCTCTACAGTGGTCAACGGGATGTCAAGACCTGGTAAGGTTCTTCGCGTTGCTTCG
AATTAAACCACATGCTCCACCGCTTGTGCGGGCCCCCGTCAATTCCTTTGAGTTTCAGTCTTGCGACCGTACTCCCCAGGCGGAGTGCTTAATGCGTTAGCTGCA
GCACTAAGGGGCGGAAACCCCCTAACACTTAGCACTCATCGTTTACGGCGTGGACTACCAGGGTATCTAATCCTGTTTGCTCCCCACGCTTTCGCGCCTCAGTGT
CAGTTACAGACCAGATAGTCGCCTTCGCCACTGGTGTTCCTCCAAATCTCTACGCATTTCACCGCTACACTTGGAATTCCACTATCCTCTTCTGCACTCAAGTCTCC
CAGTTTCCAATGACCCTCCACGGTTGAGCCGTGGGCTTTCACATCAGACTTAAGAAACCACCTGCGCGCGCTTTACGCCCAATAATTCCGGACAACGCTTGCCAC
CTACGTATTACCGCGGCTGCTGGCACGTAGTTAGCCGTGGCTTTCTAATAAAGTACCGTCAAGGTACAGCGAGATACTACTGTACGTTGTTTCTTCGCNTTACTA
CCAGAGCTTTTACGAAGTCCNNANACNNTGCATNNCCTCACGCGACGNNGCCTGCAATCATGCTTTCGNCCATNGNNNNNNNATTGNNNNACTGACCTGCCT
NCCNGGGNCGCGANNTTCNGGGGNNCACAGANNGAN

> *Bacillus cereus* D3C1
CCTANCTGTCCCCTTAGGCGGCTGGCTCCAAAAGGTTACCCCACCGACTTCGGGTGTTACAAACTCTCGTGGTGTGACGGGCGGTGTGTACAAGGCCCGGGAAC
GTATTCACCGCGCCATGCTGATCCGCGATTACTACCGATTCCGGCTTCATGTAGGCGATTTGCAACCGACAATCCAACCTGAAAACGGTTTGTTGAAATGGCTTA
CCCCCGCGGGTCCGCTGCCCCTTGTTCTGCCCTCTGTTGCACGTGTGCTGCCCACGTCATAAGGGGCATGATGAATTGTCGTCATCCCCACCTTCCTCCGGCTCCT
ACGGGAGGCTCACCTTAGTGAGCCTCCCCAATGGACGAAAGTCAAAAAAAACCACGGCTCGTGCCGGTTGAAAGACTTAACCTCCTAAAACTCTGCTGTTACGG
AAGAACAGCTGCCAGTTGATGAAGCTGTGATCTTGAAGGAAACTCACTACATAGCCGGTTGTAACTACATGCCAGGACCCGGTAAGGTTCGTCGCTTCCAAGCG
TTTTACGGAATTAGCGCCAGTAAAGGGCCCGCCCCCGGTCACTTAATTCGGATGTGAAAGCCCACGGCTCAACCGCGGGGGGTCATTGGAAACGGGGTGACTT
GAGTGCTGAAGAGGAAAGTGGCTCTCCATGTGAAGACGCGTCATGCGTAGAGAGATGGAGGAACACCCAGTGGCGAAGTCTACTCTCTGGTCTGTCACTGACA
CTGAGGCGCGAAAGCGTGGAGAGTCAACCGGGACTACTGACGTTGCTAGTCCACGCCGTAAACGATGAGTGCTAAGTGTGTAGAGGGTCTTCGCCCTTTAGCG
CTCAAGTTCTCCGCAGTTAAGCACTGCCGCCCTGACGAGTACAGGCCGGCAACGTCTGACACCCAGACGTTAAATAGAACGGACGGTGCGCGCGCGTCTACGG
CCGAATAGTGTCCTTGAATTACGGATGNANGCGTACGNCTTTAACGCAGGNCTGTTGAGCAATCGGTACNGTACAGACCGGTGANNNNACAGGTCTTTGCTAC
TNCGGAAGCAGCAGTTGNCNAAGGTCCGAACTNCANCGNNNNNNCNTCACGCTNNNNNNNNNANNNGACNNCCNGAGAGGCCCNGNGCATCGAGTCCAAN
NNCCGGNNTGTNANTCCTCACAG

> *Lysinibacillus YS11* D3C2
CNNANCTTCCACCTTCNGCGGCTGGCTCCAAAAGGTTACCTCACCGACTTCGGGTGTTACAAACTCTCGTGGTGTGACGGGCGGTGTGTACAAGGCCCGGGAA
CGTATTCACCGCGGCATGCTGATCCGCGATTACTAGCGATTCCGGCTTCATGTAGGCGAGTTGCAGCCTACAATCCGAACTGAGAACGACTTTATCGGATTAGCT
CCCTCTCGCGAGTTGGCAACCGTTTGTATCGTCCATTGTAGCACGTGTGTAGCCCAGGTCATAAGGGGCATGATGATTTGACGTCATCCCCACCTTCCTCCGGTTT
GTCACCGGCAGTCACCTTAGAGTGCCCAACTAAATGATGGCAACTAAGATCAAGGGTTGCGCTCGTTGCGGGACTTAACCCAACATCTCACGACACGAGCTGAC
GACAACCATGCACCACCTGTCACCGTTGCCCCCGAAGGGGAAACTATATCTCTACAGTGGTCAACGGGATGTCAAGACCTGGTAAGGTTCTTCGCGTTGCTTCG
AATTAAACCACATGCTCCACCGCTTGTGCGGGCCCCCGTCAATTCCTTTGAGTTTCAGTCTTGCGACCGTACTCCCCAAGCGGAGTGCTTAATGCGTTAACTGCAG
CACTAAGGAGCGGAAACCCCCTAACACTTAGCACTCATCGTTTACTACAGTGGACTACGAACGTATTTTGTCATGTTGGCTGCTCTCGCCTGTTTTGCCTCATTGT
GGCCTATAAACCAGATAGATCGCCTTCGTCACTGATGTTGGTCCTCCCAACTCCGTATACATATTAACTGCTTACTGGTTGGGAGTTTCTCCACCCTCTTTACGGC
AGTAAGTACCCCCATTTTTTCACTCCACCCCGGCAAGGGTACCGAGCGCAGGGCCCTTTACACACCAAAGTAAAAGGACACGCCGCTGCACGCACTCGTAGCCA
CCATTGTTGTTCTAAAATAGCATCTCGCCCCATATAATCTTTACCCGCGGTCCTGCGAGATCCTGGTTTATAACCCTGGGTGAAATATTATAAATAATNCCCCCCG
TCGANGCCAACAGCCTNAACTAGCTAGGNTGACCACTGTTGTTTCCTCCTCCCATTACCAGCAGTNAGTNAATAAACGNAAANCGNATTATTCCCCTCTCTNNN
NACTCACCACGCGCCGAGNCGNNNNNCTNCCATACNAGTTGNNNCACNNNNNNNNNCNNNAGTANGTGNNGNGAGNANANNNN

> *Bacillus amyloliquefaciens* D3C4
NCCCNATCTGTCCACCTTCGGCGGCTGGCTCCNTAAAAGGTTACACTCACCGACTTCGGGTGTTACAAACTCTCGTGGTGTGACGGGCGGTGTGTACACAGGCC
CGGGAACGTATTCACCGCGGCATGCTGATCCGCGATTACTAGCGATTCCAGCTTCACGCAGTCGAGTTGCAGACTGCGATCCGAACTGAGAACAGATTTGTGGG
ATTGGCTTAACCTCGCGGTTTCGCTGCCCTTTGTTCTGTCCATTGTAGCACGTGTGTAGCCCAGGTCATAAGGGGCATGATGATTTGACGTCATCCCCACCTTCCT
CCGGTTTGTCACCGGCAGTCACCTTAGAGTGCCCAACTGAATGCTGGCAACTAAGATCAAGGGTTGCGCTCGTTGCGGGACTTAACCCAACATCTCACGACACG
AGCTGACGACAACCATGCACCACCTGTCACTCTGCCCCCGAAGGGGACGTCCTATCTCTAGGATTGTCAGAGGATGTCAAGACCTGGTAAGGTTCTTCGCGTTG
CTTCGAATTAAACCACATGCTCCACCGCTTGTGCGGGCCCCCGTCAATTCCTTTGAGTTTCAGTCTTGCGACCGTACTCCCCAGGCGGAGTGCTTAATGCGTTAGC
TGCAGCACTAAGGGGCGGAAACCCCCTAACACTTAGCACTCATCGTTTACGGCGTGGACTACCAGGGTATCTAATCCTGTTCGCTCCCCACGCTTTCGCTCCTCA
GCGTCAGTTACAGACCAGAGAGTCGCCTTCGCCACTGGTGTTCCTCCACATCTCTACGCATTTCACCGCTACACGTGGAATTCCACTCTCCTCTTCTGCACTCAAG
TTCCCCAGTTTCCAATGACCCTCCCCGGTTGAGCCGGGGGCTTTCACATCAGACTTAAGAAACCGCCTGCGAGCCCTTTACGCCCAATAATTCCGGACAACGCTT
GCCACCTACGTATTACCGCGGCTGCTGGCACGTAGTTAGCCGTGGCTTTCTGGTTTAGGTACCGTCAAGGTGCCGCCCTATTTGAACGGCACTTTGTCTNCCTAC
ACAGAGCTTTTACGATCCGAANNNNCATTCANTCACGCGNNNNCTCNGTCNGGACCTTTCGTCATGCNNNNANNNNNACTGCTGCCNNCGGTNAGNNTCTG
GNNNGNNNTTCNTNA

> *Lysinibacillus sp. YS11* D4A1
CNNANCTATCCACCTTCGGCGGCTGGCTCCAAAAGGTTACCTCACCGACTTCGGGTGTTACAAACTCTCGTGGTGTGACGGGCGGTGTGTACAAGGCCCGGGA
ACGTATTCACCGCGGCATGCTGATCCGCGATTACTAGCGATTCCGGCTTCATGTAGGCGAGTTGCAGCCTACAATCCGAACTGAGAACGACTTTATCGGATTAGC
TCCCTCTCGCGAGTTGGCAACCGTTTGTATCGTCCATTGTAGCACGTGTGTAGCCCAGGTCATAAGGGGCATGATGATTTGACGTCATCCCCACCTTCCTCCGGTT

TGTCACCGGCAGTCACCTTAGAGTGCCCAACTAAATGATGGCAACTAAGATCAAGGGTTGCGCTCGTTGCGGGACTTAACCCAACATCTCACGACACGAGCTGA
CGACAACCATGCACCACCTGTCACCGTTGCCCCCGAAGGGGAAACTATATCTCTACAGTGGTCAACGGGATGTCAAGACCTGGTAAGGTTCTTCGCGTTGCTTC
GAATTAAACCACATGCTCCACCGCTTGTGCGGGCCCCCGTCAATTCCTTTGAGTTTCAGTCTTGCGACCGTACTCCCCAGGCGGAGTGCTTAATGCGTTAACTGC
AGCACTAAGGGGCGGAAACCCCCTAACACTTAGCACTCATCGTTTACTGCGTGGACTACCAAGGTATCTTATCCTGTTTGCTCCCCACGCTTTCTCGCCTCAGTGT
CGGCTACAAACCAGATAGTCGCCTTCGTTACTGGTGTTGCTCCAAATCACTACGCATTCTATCACTGCTATTGGTTGTACTCTATCCTCCTCTTTACTGCAGTCAGA
CANCTTATTATCACTCGACCCTGGTTGAGTCCGAGCGCTAGCCTATACACACTAAAAGATAATCAACGGAGCTCGCTAGCAAGCTGTAGGTCACTATGATGATCT
NNAACTAGCGATNTGCCGCCATAACTATTACCTGCGTCTTGCGTGNNNNGTATGTACCCNNGGTANNTTANTAAGTTTGCCACCGTGCNGGGCANACGCCNN
GNATNGNNNNNNNTTGTTNCNNNNNACTAGAANAGTTTGANACGGNNNNNTTCNNNCGNNNNGCTTGCTCTATAAGNNTTNGCACTATNNNNANAN

> ***Lysinibacillus sp. YS11*** **D4A2**
NCCTANCTATCCACCTTCGGCGGCTGGCTCCAAAAGGTTACCTCACCGACTTCGGGTGTTACAAACTCTCGTGGTGTGACGGGCGGTGTGTACAAGGCCCGGGA
ACGTATTCACCGCGGCATGCTGATCCGCGATTACTAGCGATTCCGGCTTCATGTAGGCGAGTTGCAGCCTACAATCCGAACTGAGAACGACTTTATCGGATTAGC
TCCCTCTCGCGAGTTGGCAACCGTTTGTATCGTCCATTGTAGCACGTGTGTAGCCCAGGTCATAAGGGGCATGATGATTTGACGTCATCCCCACCTTCCTCCGGTT
TGTCACCGGCAGTCACCTTAGAGTGCCCAACTAAATGATGGCAACTAAGATCAAGGGTTGCGCTCGTTGCGGGACTTAACCCAACATCTCACGACACGAGCTGA
CGACAACCATGCACCACCTGTCACCGTTGCCCCCGAAGGGGAAACTATATCTCTACAGTGGTCAACGGGATGTCAAGACCTGGTAAGGTTCTTCGCGTTGCTTC
GAATTAAACCACATGCTCCACCGCTTGTGCGGGCCCCCGTCAATTCCTTTGAGTTTCAGTCTTGCGACCGTACTCCCCAGGCGGAGTGCTTAATGCGTTAGCTGC
AGCACTAAGGGGCGGAAACCCCCTAACACTTAGCACTCATCGTTTACGGCGTGGACTACCAGGGTATCTAATCCTGTTTGCTCCCCACGCTTTCGCGCCTCAGTG
TCAGTTACAGACCAGATAGTCGCCTTCGCCACTGGTGTTCCTCCAAATCTCTACGCATTTCACCGCTACACTTGGAATTCCACTATCCTCTTCTGCACTCAAGTCTC
CCAGTTTCCAATGACCCTCCACGGTTGAGTCCGTGCGCTTTCACATCACACTTTAAGAAACCAACCTGCGCGCGCTTTACGCGCAATAATTACGTGTACAACGCAT
TGCCACCCTACGCTATTAAACGCTTGACTGCTGGCTACGTCGTTTAGTCTGTGGCATTTGGTAATATANATACTGTGCNACGGTACNNNGGAGACTANNACTGT
AGCTTAGTTNATGCNNTNNCATCCNNNCTATTACGGAAGTCGAAAAGNACNTCATNACTCCCNCAGACGNTNCNNCAGTCTGNNATTCNACGACTTTNNNTN
ANNNNNNNCTGAACTGCNTAGCGCGNNCGCGACNNCGGAGNNNNAN

> ***Lysinibacillus sp. YS11*** **D4A3**
ACNNANCTATCCACCTTCGGCGGCTGGCTCCNAAAGGTTACCTCACCGACTTCGGGTGTTACAAACTCTCGTGGTGTGACGGGCGGTGTGTACAAGGCCCGGG
AACGTATTCACCGCGGCATGCTGATCCGCGATTACTAGCGATTCCGGCTTCATGTAGGCGAGTTGCAGCCTACAATCCGAACTGAGAACGACTTTATCGGATTA
GCTCCCTCTCGCGAGTTGGCAACCGTTTGTATCGTCCATTGTAGCACGTGTGTAGCCCAGGTCATAAGGGGCATGATGATTTGACGTCATCCCCACCTTCCTCCG
GTTTGTCACCGGCAGTCACCTTAGAGTGCCCAACTAAATGATGGCAACTAAGATCAAGGGTTGCGCTCGTTGCGGGACTTAACCCAACATCTCACGACACGAGC
TGACGACAACCATGCACCACCTGTCACCGTTGCCCCCGAAGGGGAAACTATATCTCTACAGTGGTCAACGGGATGTCAAGACCTGGTAAGGTTCTTCGCGTTGC
TTCGAATTAAACCACATGCTCCACCGCTTGTGCGGGCCCCCGTCAATTCCTTTGAGTTTCAGTCTTGCGACCGTACTCCCCAGGCGGAGTGCTTAATGCGTTAGCT
GCAGCACTAAGGGGCGGAAACCCCCTAACACTTAGCACTCATCGTTTACGGCGTGGACTACCAGGGTATCTAATCCTGTTTGCTCCCCACGCTTTCGCGCCTCAG
TGTCAGTTACAGACCAGATAGTCGCCTTCGCCACTGGTGTTCCTCCAAATCTCTACGCATTTCACCGCTACACTTGGAATTCCACTATCCTCTTCTGCACTCAAGTC
TCCCAGTTTCCAATGACCCTCCACGGTTGAGCCGTGCGCTTTCACATCACACTTAAGAAACCAACCTGCGCGCGCTTCTACGCCCAATCAATTCCGGACAACGCTT
TGCCATCTACGTATTACACGCTGACTGCTGGCACGTAGTTTAGCATGTGGCCTTTNTAATAAATGTACCTGTCCACGGTACAGGCGCAGACTNGCTACTGTANTN
NNCATGCNNNTACNCCCNNNNTNTNNCGNNNNNGAAANTNNNTNCATAACCTCCCNNCGACGTAGCTCGAGTCTTGNNTCANCNNNCTTNNGNANNNNC
GNNNCAGACTTGCATAGCNNGTAGNGCGANNTNNNNNNNN

> ***Lysinibacillus sp. YS11*** **D4B1**
NCNNNNCTATCCACCTTCGGCGGCTGGCTCCAAAAGGTTACCTCACCGACTTCGGGTGTTACAAACTCTCGTGGTGTGACGGGCGGTGTGTACAAGGCCCGGG
AACGTATTCACCGCGGCATGCTGATCCGCGATTACTAGCGATTCCGGCTTCATGTAGGCGAGTTGCAGCCTACAATCCGAACTGAGAACGACTTTATCGGATTA
GCTCCCTCTCGCGAGTTGGCAACCGTTTGTATCGTCCATTGTAGCACGTGTGTAGCCCAGGTCATAAGGGGCATGATGATTTGACGTCATCCCCACCTTCCTCCG
GTTTGTCACCGGCAGTCACCTTAGAGTGCCCAACTAAATGATGGCAACTAAGATCAAGGGTTGCGCTCGTTGCGGGACTTAACCCAACATCTCACGACACGAGC
TGACGACAACCATGCACCACCTGTCACCGTTGCCCCCGAAGGGGAAACTATATCTCTACAGTGGTCAACGGGATGTCAAGACCTGGTAAGGTTCTTCGCGTTGC
TTCGAATTAAACCACATGCTCCACCGCTTGTGCGGGCCCCCGTCAATTCCTTTGAGTTTCAGTCTTGCGACCGTACTCCCCAGGCGGAGTGCTTAATGCGTTAGCT
GCAGCACTAAGGGGCGGAAACCCCCTAACACTTAGCACTCATCGTTTACGGCGTGGACTACCAGGGTATCTAATCCTGTTTGCTCCCCACGCTTTCGCGCCTCAG
TGTCAGTTACAGACCAGATAGTCGCCTTCGCCACTGGTGTTCCTCCAAATCTCTACGCATTTCACCGCTACACTTGGTATTCCACTATCCTCTTCTGCACTCAAGTC
TCCCAGTTTCCAATGACCCTCCACGGTTGAGCCGTGCGCTTTCACATCAGACTTAAGAAACCAACCTGCGCGCGCTTTACGCTCAATAATATCGTGACAACGCTTG
CCACCTACGTATTACCTGCTGACTGCTGGCTCGTNGTTAGTCTGTGGCATTTGTAAATATGTACNAGTGCCACGTACAGAGAAATACGCTACGTGTANTGNNNA
TGNTAACTACCNNNNCTNNTAGGNAGGTCNNGAANNNNNNNTACNNNCGCNACGTTNGCGCNCNGNNNTGCATTCANNNCGNNNGNTTNNNNNNNNNC
GTGAACGCACTCCNTNNCGNGGTANGAGCNTCCGNAGACNAGATGTC

> ***Lysinibacillus sp. YS11*** **D4B2**
CCTANNTATCCACCTTCGGCGGCTGGCTCCAAAAGGTTACCTCACCGACTTCGGGTGTTACAAACTCTCGTGGTGTGACGGGCGGTGTGTACAAGGCCCGGGAA
CGTATTCACCGCGGCATGCTGATCCGCGATTACTAGCGATTCCGGCTTCATGTAGGCGAGTTGCAGCCTACAATCCGAACTGAGAACGACTTTATCGGATTAGCT
CCCTCTCGCGAGTTGGCAACCGTTTGTATCGTCCATTGTAGCACGTGTGTAGCCCAGGTCATAAGGGGCATGATGATTTGACGTCATCCCCACCTTCCTCCGGTTT
GTCACCGGCAGTCACCTTAGAGTGCCCAACTAAATGATGGCAACTAAGATCAAGGGTTGCGCTCGTTGCGGCACTTATCCCAACATCTCACGACACGAGCTGAC
GACAACCATGCACCACCTGTCACCGTTGCCCCCGAAGGGGAAACTATATCTCTATAGTGGTCAACGGGATGTCAAGACCTGGTAAGGTTCTTCCCGTTGCTTTGA
ATTAAAAACATGCTCCACCGCTTGTGCGGGCCCCCGTCAATTACTTTTAGTTTTTATCTTGCGGGCGTACTCCCCAGGAGGAGTGCTTAATAAGTTTACTTGGCAC
TACTAGAGCGGAGGGACCTCCCAATTATTTACTTCTCATTTGTTTACGGTGGTCTACGACGAACATTGTCATCTT

> ***Lysinibacillus sp. YS11*** **D4B3**
CNNNNCTATCCACCTTCGGCGGCTGGCTCCAAAAGGTTACCTCACCGACTTCGGGTGTTACAAACTCTCGTGGTGTGACGGGCGGTGTGTACAAGGCCCGGGA
ACGTATTCACCGCGGCATGCTGATCCGCGATTACTAGCGATTCCGGCTTCATGTAGGCGAGTTGCAGCCTACAATCCGAACTGAGAACGACTTTATCGGATTAGC
TCCCTCTCGCGAGTTGGCAACCGTTTGTATCGTCCATTGTAGCACGTGTGTAGCCCAGGTCATAAGGGGCATGATGATTTGACGTCATCCCCACCTTCCTCCGGTT
TGTCACCGGCAGTCACCTTAGAGTGCCCAACTAAATGATGGCAACTAAGATCAAGGGTTGCGCTCGTTGCGGGACTTAACCCAACATCTCACGACACGAGCTGA
CGACAACCATGCACCACCTGTCACCGTTGCCCCCGAAGGGGAAACTATATCTCTACAGTGGTCAACGGGATGTCAAGACCTGGTAAGGTTCTTCGCGTTGCTTC

GAATTAAACCACATGCTCCACCGCTTGTGCGGGCCCCCGTCAATTCCTTTGAGTTTCAGTCTTGCGACCGTACTCCCCAGGCGGAGTGCTTAATGCGTTAGCTGC
AGCACTAAGGGGCGGAAACCCCCTAACACTTAGCACTCATCGTTTACGGCGTGGACTACCAAGGTATCTAATCCTGTTTGCTCCCCACGCTTTCGCGCCTCAGTG
TCAGTTACAGACCAGATAGTCGCCTTCGCCACTGGTGTTCCTCCAAATCTCTACGCATTTCATCAGTGCACTTGGTAGTTGGACTATCCTCTTCTGCACTCAAGTCT
CTCAGTTTTTAATGACTCCACCACGGTTGAGCCGTGCGCTGTCACTATCACACTTAAATAAACAGACTGCGCGCGCTCTACGCTTCAGTCACTTCGGGACATCAGC
TTAGCATCCTACGCTATAAAATNTTGACTGCGTGTCTANGTGNTNNGATTGTACACTGGTATATATAGTACTGTGCACGTACGAGGCAATAGNTAGAGTAGGTA
GNGCATTTGCTTGACNNNNNNNNCTAGAGNAAGTTCNGAAGGAACNNNNNANTTCCNNNACGTAGCGCNNGCNTTGCTNTCATNCANGTTCNNNNNCNNNT
NNNNNACNNNANNNNNNNNNACNNNGGANAGGAGC

> *Bacillus cereus* D4C1
ACCATATCTGTCCACCTTAGGCGGCTGGCTCCAAAAAGGTTACCCCACCGACTTCGGGTGTTACAAACTCTCGTGGTGTGACGGGCGGTGTGTACAAGGCCCGG
GAACGTATTCACCGCGGCATGCTGATCCGCGATTACTAGCGATTCCAGCTTCATGTAGGCGAGTTGCAGCCTACAATCCGAACTGAGAACGGTTTTATGAGATT
AGCTCCACCTCGCGGTCTTGCAGCTCTTTGTACCGTCCATTGTAGCACGTGTGTAGCCCAGGTCATAAGGGGCATGATGATTTGACGTCATCCCCACCTTCCTCCG
GTTTGTCACCGGCAGTCACCTTAGAGTGCCCAACTTAATGATGGCAACTAAGATCAAGGGTTGCGCTCGTTGCGGGACTTAACCCAACATCTCACGACACGAGC
TGACGACAACCATGCACCACCTGTCACTCTGCTCCCGAAGGAGAAGCCCTATCTCTAGGGTTTTCAGAGGATGTCAAGACCTGGTAAGGTTCTTCGCGTTGCTTC
GAATTAAACCACATGCTCCACCGCTTGTGCGGGCCCCCGTCAATTCCTTTGAGTTTCAGCCTTGCGGCCGTACTCCCCAGGCGGAGTGCTTAATGCGTTAACTTCA
GCACTAAAGGGCGGAAACCCTCTAACACTTAGCACTCATCGTTTACGGCGTGGACTACCAGGGTATCTAATCCTGTTTGCTCCCCACGCTTTCGCGCCTCAGTGT
CAGTTACAGACCAGAAAGTCGCCTTCGCCACTGGTGTTCCTCCATATCTCTACGCATTTCACCGCTACACATGGAATTCCACTTTCCTCTTCTGCACTCAAGTCTCC
CAGTTTCCAATGACCCTCCACGGTTGAGCCGTGGGCTTTCACATCAGACTTAAGAAACCACCTGCGCGCGCTTTACGCCCAATAATTCCGGATAACGCTTGCCAC
CTACGTATTACCGCGGCTGCTGGCACGTAGTTAGCCGTGGACTTTCTGGTTAGGTACCGTCAAGGTGCCAGCTTATTCAACTAGCACTTGTTCTTCCCTAACNNN
GAGTTTTACGACCCGAAGCCATCATCACTCACGCGCGTGCTCGTCNGANTTCGTCCATGCGNNNANNNTACTGCTGNNCCGTAGGANTCTGGNCGNGTTCTCA
GTNCCCAGTGTTGGNNNN

> *Bacillus cereus* D4C2
NCCATATCTGTCCACCTTAGGCGGCTGGCTCCAAAAAGGTTACCCCACCGACTTCGGGTGTTACAAACTCTCGTGGTGTGACGGGCGGTGTGTACAAGGCCCGG
GAACGTATTCACCGCGGCATGCTGATCCGCGATTACTAGCGATTCCAGCTTCATGTAGGCGAGTTGCAGCCTACAATCCGAACTGAGAACGGTTTTATGAGATT
AGCTCCACCTCGCGGTCTTGCAGCTCTTTGTACCGTCCATTGTAGCACGTGTGTAGCCCAGGTCATAAGGGGCATGATGATTTGACGTCATCCCCACCTTCCTCCG
GTTTGTCACCGGCAGTCACCTTAGAGTGCCCAACTTAATGATGGCAACTAAGAGCAAGGGTTGCGCTCGTTGCGGGACTTAACCCAACATCTCACGACACGAGC
TGACGACAACCATGCACCACCTGTCACTCTGCTCCCGAAGGAGAAGCCCTATCTCTAGGGTTTTCAGAGGATGTCAAGACCTGGTAAGGTTCTTCGCGTTGCTTC
GAATTAAACCACATGCTCCACCGCTTGTGCGGGCCCCCGTCAATTCCTTTGAGTTTCAGCCTTGCGGCCGTACTCCCCAGGCGGAGTGCTTAATGCGTTAACTTCA
GCACTAAAGGGCGGAAACCCTCTAACACTTAGCACTCATCGTTTACGGCGTGGACTACCAGGGTATCTAATCCTGTTTGCTCCCCACGCTTTCGCGCCTCAGTGT
CAGTTACAGACCAGAAAGTCGCCTTCGCCACTGGTGTTCCTCCATATCTCTACGCATTTCACCGCTACACATGGAATTCCACTTTCCTCTTCTGCACTCAAGTCTCC
CAGTTTCCAATGACCCTCCACGGTTGAGCCGTGGGCTTTCACATCAGACTTAAGAAACCACCTGCGCGCGCTTTACGCCCAATAATTCCGGATAACGCTTGCCAC
CTACGTATTACCGCGGCTGCTGGCACGTAGTTAGCCGTGACTTTCTGGTTAGGTACCGTCAAGGTGCCAGCTTATTCAACTAGCACTTGTTCTCCTACAACAGAG
TTTTACGACCGAAAGCGTCATCANTCACGCGNGTGCTCGTCNGANTTCGTCATTGCGNNANNNTACTGCTGGCNNCNGTNNNNTCTGGACNGTGNTCTCAGT
CNNNNGGTGNNNNNTN

> *Bacillus sp. YS11* D4C3
CCATACGCTGTCCNCCTTAGTCGGCTGGCTCCAAAAGGTTACCCCACCGACTTCGGGTGTTACAAACTCTCGTGGTGTGACGGGCGGTGTGTACAAGGCCCGGG
AACGTATTCACCGCGGCATGCTGATCCGCGATTACTAACGATTCCAGCTTCATGTAGGCGAGTTGCAGCCTACCATCCGAACTGAAAACGGTTTTATGAAATTAG
CTCCCCTCGCGGTCTTGCATCTCTTTGTACCGTCCATTGTAGCACGTGTGTAGCCCAGGTCATAAGGGGCATGATGATTTGACGTCATCCCCACCTTCCTCCGGTT
TGTCACCGGCAGTCACCTTAGAGTGCCCAACTTAATGATGGCAACTAAGAGCAAGGGTTGCGCTCGTTGCGGGACTTAACCCAACATCTCACGACACGAGCTGA
CGACAACCATGCACCACCTGTCACTCTGCTCCCGAAGGAGAAACCCTATCTCTAGGGTTTTCATAGGATGTGGCAAACAGCTAAGGTTCTTCGCGTTGGTTCTAC
TTAAACCACATGCACTACCCCTTGTGCGGGGCGCCCCTGGTTACTTTTATTTGATTTAAAAACCTCCCGGCCTCCCCCGGGGAGGTGATTAGTAAATTGGCTAACT
TCATCGCCGAAGGGCACACACCCATCTCAATGTTAACACTTAACTTGCAAAGCGTGACCAACGAACATAACTAGCGATGGTGGCTGTCCACGCCTGTAAACCACT
AATGTTAGCTGAAAGCGTGGAGAGCACCCTGGGATCATAGACCGTTGTTCGTTAAACTCTGCAAATTACGAGTGCTAAGTTGTAAGATGCAATTCCCCCCCTTTA
GGGCTGAAGGTTACGCATTTACGAAGGCCGCTCTGGGGGATTAACGGCCAAGCAGTTTAAAAAACAAAATATAAAGAGACCCGCGCCCTCCACAATTACTGCA
AAAATGTTGGCTTAAATTCGGTTTGACCACTTAGAAATCTATCCGCGGTTCTTGGCATCGCTATGAAAACGTGTACATAAATGGGGTCAGCTACTGTCAGAGCAG
AATGGACAAGACAGATACATTATTTGTTCGTCACGCTCATCACTGGAAGTATTGCGAGCCTGTAAAGTTGCCTTAATACATATCGCCACACCGGGTTGTGGTTTC
TTTATGNNTTGAACATTTTTNTTTNCACTTGGGGGGAAGAAAATAANNNGNNAAANNTGGCTCGCGCNNTTCNNNNAAAACNNNNNNTANNATAANNNNT
NGGTGAGTCNTGTACTAAGAAAACT

> *Bacillus cereus* D4C4
CCATATCTGTCCACCTTAGGCGGCTGGCTCCAAAAAGGTTACCCCACCGACTTCGGGTGTTACAAACTCTCGTGGTGTGACGGGCGGTGTGTACAAGGCCCGGG
AACGTATTCACCGCGGCATGCTGATCCGCGATTACTAGCGATTCCAGCTTCATGTAGGCGAGTTGCAGCCTACAATCCGAACTGAGAACGGTTTTATGAGATTA
GCTCCACCTCGCGGTCTTGCAGCTCTTTGTACCGTCCATTGTAGCACGTGTGTAGCCCAGGTCATAAGGGGCATGATGATTTGACGTCATCCCCACCTTCCTCCGG
TTTGTCACCGGCAGTCACCTTAGAGTGCCCAACTTAATGATGGCAACTAAGATCAAGGGTTGCGCTCGTTGCGGGACTTAACCCAACATCTCACGACACGAGCT
GACGACAACCATGCACCACCTGTCACTCTGCTCCCGAAGGAGAAGCCCTATCTCTAGGGTTTTCAGAGGATGTCAAGACCTGGTAAGGTTCTTCGCGTTGCTTCG
AATTAAACCACATGCTCCACCGCTTGTGCGGGCCCCCGTCAATTCCTTTGAGTTTCAGCCTTGCGGCCGTACTCCCCAGGCGGAGTGCTTAATGCGTTAACTTCAG
CACTAAAGGGCGGAAACCCTCTAACACTTAGCACTCATCGTTTACGGCGTGGACTACCAGGGTATCTAATCCTGTTTGCTCCCCACGCTTTCGCGCCTCAGTGTCA
GTTACAGACCAGAAAGTCGCCTTCGCCACTGGTGTTCCTCCATATCTCTACGCATTTCACCGCTACACATGGAATTCCACTTTCCTCTTCTGCACTCAAGTCTCCCA
GTTTCCAATGACCCTCCACGGTTGAGCCGTGGGCTTTCACATCAGACTTAAGAAACCACCTGCGCGCGCTTTACGCCCAATAATTCCGGATAACGCTTGCCACCT
ACGTATTACCGCGGCTGCTGGCACGTAGTTAGCCGTGGCTTTCTGGGTTAGGGTACCGTCAAGGTGCCAGCTTATTCAACTAGCACTTGTTCTTCCCTACANAGA
GTTTTACGACCCGAAAGCCTTCATCACTCACGCGNNTNCTCGTCNGANTTTNCGTCNATGCNNNATNCTNACTGCTGCTCCGTAGNNNCTGGACGNGGTTCTC
AGTTNNAAGNTGTTGTGNNNNN

> *Bacillus cereus* D5B1

CCNANCTGTCCACCTTAGGCGGCTGGCTCCAAAAAGGTTACCCCACCGACTTCGGGTGTTACAAACTCTCGTGGTGTGACGGGCGGTGTGTACAAGGCCCGGG
AACGTATTCACCGCGGCATGCTGATCCGCGATTACTAGCGATTCCAGCTTCATGTAGGCGAGTTGCAGCCTACAATCCGAACTGAGAACGGTTTTATGAGATTA
GCTCCACCTCGCGGTCTTGCAGCTCTTTGTACCGTCCATTGTAGCACGTGTGTAGCCCAGGTCATAAGGGGCATGATGATTTGACGTCATCCCCACCTTCCTCCGG
TTTGTCACCGGCAGTCACCTTAGAGTGCCCAACTTAATGATGGCAACTAAGATCAAGGGTTGCGCTCGTTGCGGGACTTAACCCAACATCTCACGACACGAGCT
GACGACAACCATGCACCACCTGTCACTCTGCTCCCGAAGGAGAAGCCCTATCTCTAGGGTTTTCAGAGGATGTCAAGACCTGGTAAGGTTCTTCGCGTTGCTTCG
AATTAAACCACATGCTCCACCGCTTGTGCGGGCCCCCGTCAATTCCTTTGAGTTTCAGCCTTGCGGCCGTACTCCCCAGGCGGAGTGCTTAATGCGTTAACTTCAG
CACTAAAGGGCGGAAACCCTCTAACACTTAGCACTCATCGTTTACGGCGTGGACTACCAGGGTATCTAATCCTGTTTGCTCCCCACGCTTTCGCGCCTCAGTGTCA
GTTACAGACCAGAAAGTCGCCTTCGCCACTGGTGTTCCTCCATATCTCTACGCATTTCACCGCTACACATGGAATTCCACTTTCCTCTTCTGCACTCAAGTCTCCCA
GTTTCCAATGACCCTCCACGGTTGAGCCGTGGGCTTTCACATCAGACTTAAGAAACCACCTGCGCGCGCTTTACGCCCAATAATTCCGGATAACGCTTGCCACCT
ACGTATTACCGCGGCTGCTGGCACGTAGTTAGCCGTGGCTTTCTGGTTTAGGTACNGTCAAGGTGCCAGCTGATTCAACTAGCACTTGTCTNCCTACNNAGAGT
TTTACGACCCGGAAAGCGTCATCACTCANGCCGCNTTGCCTCGTTCAGANTTNCGTCCATTGNNGGNANNNNNNTACTGACTGACCNNNNNNNNNNNNNGN
NCAGTGTNCTNAGTNCCNANNNTGNGTTTGN

References

1 Liu, G.-H., Liu, B., Wang, J.-P., Che, J.-M. & Li, P.-F. Reclassification of Brevibacterium frigoritolerans DSM 8801 T as Bacillus frigoritolerans comb. nov. Based on Genome Analysis. *Current microbiology* **77**, 1916-1923 (2020).

2 Jin, M. *et al.* Draft Genome Sequence of a Potential Organic Phosphorus-Degrading Bacterium Brevibacterium frigoritolerans GD44, Isolated from Radioactive Soil in Xinjiang, China. *Current Microbiology* **77**, 2896-2903 (2020).

3 Carrera, M., Zandomeni, R. O., Fitzgibbon, J. & Sagripanti, J.-L. Difference between the spore sizes of Bacillus anthracis and other Bacillus species. *Journal of Applied Microbiology* **102**, 303-312, doi:https://doi.org/10.1111/j.1365-2672.2006.03111.x (2007).

4 Weart, R. B. *et al.* A metabolic sensor governing cell size in bacteria. *Cell* **130**, 335-347, doi:10.1016/j.cell.2007.05.043 (2007).

5 Meng, D. *et al.* Rapid and simultaneous screening of pathway designs and chassis organisms, applied to engineered living materials. *Metabolic Engineering*, doi:https://doi.org/10.1016/j.ymben.2021.01.006 (2021).

6 Kamble, A. & Singh, H. Different Methods of Soil DNA Extraction. *Bio-protocol* **10**, e3521, doi:10.21769/BioProtoc.3521 (2020).

7 Maciel, B. *et al.* Simple DNA extraction protocol for a 16S rDNA study of bacterial diversity in tropical landfarm soil used for bioremediation of oil waste. *Genet Mol Res* **8**, 375-388 (2009).

8 Zhang, J., Kobert, K., Flouri, T. & Stamatakis, A. PEAR: a fast and accurate Illumina Paired-End reAd mergeR. *Bioinformatics* **30**, 614-620, doi:10.1093/bioinformatics/btt593 (2014).

9 Bolyen, E. *et al.* Reproducible, interactive, scalable and extensible microbiome data science using QIIME 2. *Nature biotechnology* **37**, 852-857 (2019).

10 Callahan, B. J. *et al.* DADA2: high-resolution sample inference from Illumina amplicon data. *Nature methods* **13**, 581-583 (2016).

11 Wickham, H., Chang, W. & Wickham, M. H. Package 'ggplot2'. *Create Elegant Data Visualisations Using the Grammar of Graphics. Version* **2**, 1-189 (2016).

12 Yoon, S.-H. *et al.* Introducing EzBioCloud: a taxonomically united database of 16S rRNA gene sequences and whole-genome assemblies. *Int J Syst Evol Microbiol* **67**, 1613-1617, doi:10.1099/ijsem.0.001755 (2017).

13 Dereeper, A. *et al.* Phylogeny.fr: robust phylogenetic analysis for the non-specialist. *Nucleic acids research* **36**, W465-W469, doi:10.1093/nar/gkn180 (2008).

14 Wood, D. E., Lu, J. & Langmead, B. Improved metagenomic analysis with Kraken 2. *Genome Biology* **20**, 257, doi:10.1186/s13059-019-1891-0 (2019).

15 Prjibelski, A., Antipov, D., Meleshko, D., Lapidus, A. & Korobeynikov, A. Using SPAdes De Novo Assembler. *Current Protocols in Bioinformatics* **70**, e102, doi:https://doi.org/10.1002/cpbi.102 (2020).

16 Nurk, S., Meleshko, D., Korobeynikov, A. & Pevzner, P. A. metaSPAdes: a new versatile metagenomic assembler. *Genome Res* **27**, 824-834, doi:10.1101/gr.213959.116 (2017).

17 Walker, B. J. *et al.* Pilon: An Integrated Tool for Comprehensive Microbial Variant Detection and Genome Assembly Improvement. *PLOS ONE* **9**, e112963, doi:10.1371/journal.pone.0112963 (2014).

18 Delcher, A. L., Salzberg, S. L. & Phillippy, A. M. Using MUMmer to identify similar regions in large sequence sets. *Curr Protoc Bioinformatics* **Chapter 10**, Unit 10.13, doi:10.1002/0471250953.bi1003s00 (2003).

19 Deatherage, D. E. & Barrick, J. E. in *Engineering and analyzing multicellular systems* 165-188 (Springer, 2014).

20 Skinner, S. O., Sepúlveda, L. A., Xu, H. & Golding, I. Measuring mRNA copy number in individual Escherichia coli cells using single-molecule fluorescent in situ hybridization. *Nat Protoc* **8**, 1100-1113, doi:10.1038/nprot.2013.066 (2013).
